# Anthocyanins: Traditional Uses, Structural and Functional Variations, Approaches to Increase Yields and Products’ Quality, Hepatoprotection, Liver Longevity, and Commercial Products

**DOI:** 10.3390/ijms23042149

**Published:** 2022-02-15

**Authors:** Hamdoon A. Mohammed, Riaz A. Khan

**Affiliations:** 1 Department of Medicinal Chemistry and Pharmacognosy, College of Pharmacy, Qassim University, Qassim 51452, Saudi Arabia; 2 Department of Pharmacognosy, Faculty of Pharmacy, Al-Azhar University, Cairo 11371, Egypt

**Keywords:** anthocyanins, traditional medicine, liver protection, hepatocellular longevity, hepatic carcinoma, anti-oxidant, anti-inflammation, NrF2, TNF-α, anthocyanins-rich extracts, anthocyanins marketed products

## Abstract

Anthocyanins are water-soluble, colored compounds of the flavonoid class, abundantly found in the fruits, leaves, roots, and other parts of the plants. The fruit berries are prime sources and exhibit different colors. The anthocyanins utility as traditional medicament for liver protection and cure, and importance as strongest plants-based anti-oxidants have conferred these plants products different biological activities. These activities include anti-inflammation, liver protective, analgesic, and anti-cancers, which have provided the anthocyanins an immense commercial value, and has impelled their chemistry, biological activity, isolation, and quality investigations as prime focus. Methods in extraction and production of anthocyanin-based products have assumed vital economic importance. Different extraction techniques in aquatic solvents mixtures, eutectic solvents, and other chemically reactive extractions including low acid concentrations-based extractions have been developed. The prophylactic and curative therapy roles of the anthocyanins, together with no reported toxicity has offered much-needed impetus and economic benefits to these classes of compounds which are commercially available. Information retrieval from various search engines, including the PubMed^®^, ScienceDirect^®^, Scopus^®^, and Google Scholar^®^, were used in the review preparation. This imparted an outlook on the anthocyanins occurrence, roles in plants, isolation-extraction, structures, biosynthetic as well as semi- and total-synthetic pathways, product quality and yields enhancements, including uses as part of traditional medicines, and uses in liver disorders, prophylactic and therapeutic applications in liver protection and longevity, liver cancer and hepatocellular carcinoma. The review also highlights the integrated approach to yields maximizations to meet the regular demands of the anthocyanins products, also as part of the extract-rich preparations together with a listing of marketed products available for human consumption as nutraceuticals/food supplements.

## 1. Perspective, Scope, and Methodology for Information Retrieval of Anthocyanins

The interest and information on anthocyanins, and its aglycone precursors, anthocyanidins, as being part of the phenolics-rich and flavonoid class of products, with exhibitions of a wide spectrum of different biological activity and broadly considered health benefits is of immense value. The strong anti-oxidant potential together with their effectiveness as anti-inflammatory, analgesic, anti-cancers, and liver protective, and diseased liver therapy activities of the flavonoids in general, and to certain extent, the anthocyanins in particular with certain biological activity, especially the antioxidant, liver protective, and anti-liver cancer have propelled the anthocyanins and the anthocyanins-rich extracts into limelight. The continued interest in structurally advanced flavonoids, and their perpetual contributions and discoveries of newer roles in plants and animal kingdoms of their biology, pharmacological actions, metabolism, physiology, plants’ protectives roles, and the intrinsic inter-relationship in plants’ survival as well as their constituents’ medicinal values have caught the attention of the scientific community. The traditional uses, and reported biological activities of the anthocyanin class of products, their consumptive implications for human health, and anthocyanin-rich plants’ extracts together with the evaluations of variant biology and their structural chemistry also formed part of the information retrieval in the scope for the anthocyanins at large. The natural availability, product types and constituents’ concentrations, products’ yields and its enhancements, enrichment in extracts, as well as methodologies adopted to locate and finger-print the anthocyanin constituents in various anthocyanins-rich plants were pursued. The general traditional uses of plants, plants rich in anthocyanins, plants providing liver protection, and prescribed in liver therapy for different ailments, plants and plants-based products in co-occurrence with the flavonoids, other polyphenols, and anthocyanins which has been found to be detrimental to liver protection, as well as causing liver toxicity were also included to provide the positive aspects of the plants-based products in liver therapy. This notion of counter activity and toxic effects is very commonly found and adopted in the traditional realm through the Materia Medica of different cultures and societies from east to west and Midwest. The anthocyanins-based marketed plants extracts, extracts rich in anthocyanins, crude and pure anthocyanins/anthocyanidins were also checked out from different information sources including the traditional sources on medicinal treatise, local herbalist practices, as well as different literature search engines, i.e., EuropePMC^®^, ScienceDirect^®^, PubMed^®^, Google Scholar^®^, Google^®^, Scopus^®^, and Scifinder^®^, including the lesser exposed search libraries and repositories of various institutions and agencies as also encountered during the searches. The review covers the aspects of anthocyanins distributions and their functional roles in plants, chemistry, structures and SAR (structure activity relationships), biotechnical, biosynthetic, as well as semi-synthetic and synthetic pathways integrated together to suggest to overcome the increasing demands for anthocyanin-based products in the market. The review also sheds light on the anthocyanin-products’ extractions and the products’ quality in terms of contents, anthocyanin contents’ quantitative analysis by spectro-chromatographic techniques, and anthocyanins-based commercialized products. The toxicity of anthocyanins as an important ingredient of the liver therapy regime was also deliberated. Nearly 27,000, 36,000, 14,900, 219,000, and 19,200,000 entries were retrieved in EuropePMC^®^, ScienceDirect^®^, PubMed^®^, Google Scholar^®^, and Google^®^ search engines, and were sub-searched and sifted for different terms as stipulated for chemical structure, biological activity, occurrence, distribution, synthesis, semi-synthesis, biosynthesis, and commercial products. The search term criteria were exhausted in different search engines and redundant as well as repeated resources were sorted out through limiting the search criteria, time-line, and the terms thereof. A ratio of nearly 5000 reviews, 5000 book chapters, and 20,000 research articles were observed as the retrieval patterns for Science Direct^®^, PubMed^®^ and Google Scholar^®^ search sites with over half of these in the last 5 years. A selection of information fitting the search criteria have been included to serve the review outline. 

## 2. Anthocyanins’ Aesthetics, and Plant Kingdom’s Distribution

The colorful world of the plant kingdom owes its beauty, attractiveness, attention, and diligence primarily to the charged and colorful flavonoid-based structures which are identified as anthocyanins. The anthocyanins are regarded as the largest, most interesting, as well as intriguing group of plants-based pigments under use by humans from very early times as colorants for foods, beverages and clothes, baits, armors, phytopharmaceuticals, colors for drawings, cave-arts, and for festivities. The anthocyanin stands for two Greek words, i.e., *anthos* for flower, and *kyaneos* for dark blue color. They are located in plant cell vacuoles, and owing to multi-colored appearances in different visible parts of the plants, including flowers, fruits, leaves, tubers, and roots, they have been in focus in various human activities including medicinal uses. The anthocyanins are appealing to humans, and attracted insects and animals in utility toward the pollination, seeds and fruits dispersal, as well as indirect carriers of plants’ species spread, conservation and natural balances [1]. The anthocyanins constitute nearly one-third of the flavonoids and are water-soluble, structurally polyphenolic in nature. They are specifically distributed in plums, cherries, and berries of several plants, have acquired different colors of purple, red, violet, pink, and blue, which indicated the apparent presence of this class of compounds in nature. The plant families of Berberidaceae, Eleaocarpaceae, Myrtaceae, Solanaceae, and Rosaceae are among the major contributors [2,3]. Several crops, including the fruits of acai, cherry, black currants, black crowberry, blueberries, blackberries, bilberry, Andean black berries, cranberry, cowberry, gojiberry, Chilean berries, European bilberry, American cranberry, mulberry, red raspberries, black raspberries, choke berries (aronia berry), boysenberry, strawberry, sourberry, bosberry, jostaberry, rabbit-eye-berry, low-bushberry, high-bushberry, half-high-bushberry, buffaloberry, skunkberry, oval-leaf-huckleberry, Canadaberry, olallieberry, juneberry, sumacberry, sloeberry, turkeyberry, huckleberry, salmonberry, saskatoonberry, maquiberry, marionberry, cloudberry, pineberry, seaberry, tayberry, coralberry, yewberry, tart cherries, Concord and Norton grapes, black plums, black corn, black beans, purple onions, red radish, red currant, red cabbage, red onions, red lettuce, red-skinned potato, broccoli, rhubarb, fennel, lettuce, brown beans, seabuckthorn, purple sweet potatoes, peach, tomato, pistachio nut, pomegranate, nectarine, apples, turnip, European and Mediterranean olives, blood orange, purple carrot, black carrot, tea, coffee beans, and black rice, etc., have been found to be rich in anthocyanins contents [4,5,6,7,8,9,10,11,12,13,14,15,16,17,18].

The anthocyanins distribution has been followed from the beginning of their discovery as pigments from plants, and information on finger-printings obtained through the chemical profiling of different extracts of several fruits and other plant parts have led the way to identify, compare, and establish the anthocyanins presence in several plant species. The use of HPLC, LC-MS [19], HPLC-DAD-ESI/MS/MS [4,20], and other different mass (MS) techniques, including soft ion bombardment, electrospray, and TOF (time of flight) techniques have contributed immensely toward the anthocyanins discovery and structure elucidations [12,21,22,23,24,25]. Among the other techniques, through established protocols of the methods, the chromatographic and spectro-analytical methods were also employed. The UV-visible spectrophotometry in conjunction with pH variability [26], mass spectrometry (MS) [27], and NMR (nuclear magnetic resonance) techniques have been utilized and anthocyanins presence have been defined at large scale [28]. The concentrations and structurally varied anthocyanins have been reported from various sources through advancements in techniques and methodology development [29]. For instance, the anthocyanins level in fresh berries (gooseberry and chokeberry) has been found varying from 0.7 to 1480 mg/100 g, while the most abundant anthocyanin-based compounds were found as the cyanidin, delphinidin, petunidin, pelargonidin, peonidin, and malvidin-based glycosylated molecules [4,30,31]. Nonetheless, the concentration of anthocyanins and their specific structural types differ in various different sources of the plant kingdom. The anthocyanins are highly affected by the temperature, light, and agronomic factors, which have been reported as major reasons for their considerable variations in the anthocyanins’ contents, as well as their structure-based variations among several fruits and vegetable types [32]. In this regards, the intersecting anthocyanins cluster distribution in the plant kingdom based on the Phenol Explorer Online Database, has been performed by Mannino et al., [33], which demonstrated the anthocyanins concentrations in different plants in ascending order from 15 mg (e.g., grape fruits, date, and red onion) to 500 mg/100 g (e.g., black chokeberry, black raspberry, and evergreen huckleberry) of the plants [33]. The exercise has provided a useful and predictive range of product types and their probabilistic as well as expected concentrations. 

## 3. Anthocyanins Roles in the Plants

The anthocyanins play beneficial role as a protective barrier for plants against high influx of light intensity, and the UV-B light [33]. The anthocyanins as antioxidant plant contents have primary defensive role in plants against various abiotic stress, including drought and high salinity conditions, as well as work against heat and light stresses. The anthocyanins are also involved in functioning and regulations of senescence, leaf temperature, osmotic balance, monosaccharides transport, and camouflage [33]. Therefore, the anthocyanins play definite part in protecting the photosynthetic apparatus of the plants from high light radiation flux [34], and avoid the damage to the plant DNA. The anthocyanins also protect against water, cold, heat, and drought stress, as well as help regulate the hemostasis of the plants [35]. Besides, the anthocyanins role in the protection of plants against different biotic stresses, e.g., microbial and insect attacks, have also been reported [36,37]. The anthocyanins contents in plants vary by the plant species and its variety, the prevalent environmental factors, plants’ growth stages, and the plant-products’ storage [38]. Anthocyanins are also accumulated over time in the plant vacuoles, thereby reaching maximum at ripening age of the fruits, and are also distributed in different plants parts, including in the autumn [39,40]. 

## 4. Chemistry of the Anthocyanins, Structures, and the Structural Variants 

**Figure 1:**
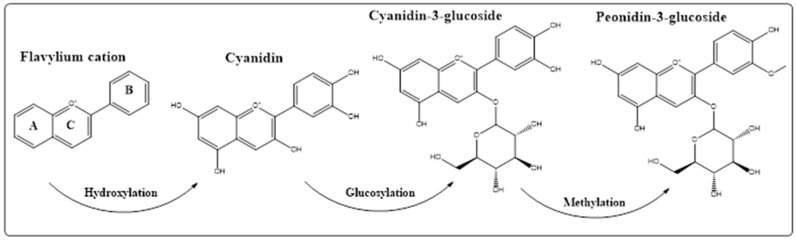
Basic skeleton and common biogenetic pathway of the anthocyanins.

Structurally, the anthocyanins are part of the flavonoid series of plants products, and have been categorized as part of C_6_-C_3_-C_6_ molecular framework. The anthocyanin products are found as the sugar-bonded counterparts (glycosides) of the anthocyanidins (aglycones) analogs [41], in which fifteen carbon atoms-based framework skeleton, flavylium cation, is arranged in three rings denoted as A, B, and C, which form the part of the basic skeleton of the anthocyanin compounds (Figure 1). The anthocyanin compounds usually have multi-hydroxylation patterns distributed along the three-ring structure of the compound, especially at the C-3 position of the ring C; C-5, C-6, and C-7 positions of the ring A; and C-3′, C-4′, and C-5′ positions of the ring B [42]. The sugar moieties are attached to the anthocyanidins’ structure through the formation of acetal linkage with one, or more of the mentioned hydroxyl groups. The most common and abundant anthocyanin glycosides are produced by the glycosylation of the 3-OH group to form the 3-O-β-glucosides derivatives, e.g., cyanidin-3-O-β-glucoside and peonidin-3-O-glucoside (Figure 1) [43]. 

The anthocyanin compounds provide different colors to the plants which are attributed to the highly resonating electrons around the flavylium ion structure [44,45]. Besides, the variety of the hydroxylation patterns of the anthocyanins in different positions along the three rings (rings A, B, and C) of the compounds, and also, the types and positions of the glycosylation, and the carboxylates attached to the sugar moieties, contribute to the diversification of huge varieties of the identified anthocyanin compounds, and their colors [41].

**Figure 2:**
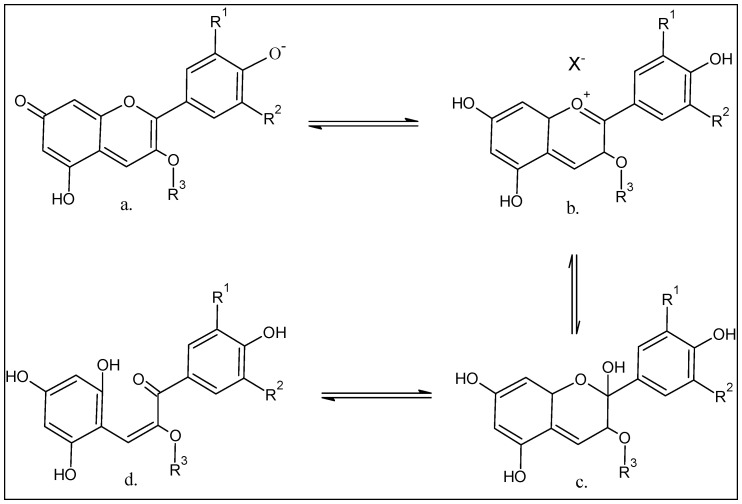
Effects of pH conditions on colors of anthocyanins: R^1^ = R^2^ = OH/O-CH_3_/OC(O)CH_3_; R^3^ = glycoside; (**a**) quinonoid base (pH 8–10) changes to, (**b**) flavylium cation (pH < 2, red to orange color), (**c**) carbinol pseudo-base (pH range 3–6, colorless), (**d**) chalcone pseudo-base (high pH, pale yellow to colorless). The X^−^ refer to the anionic entity, usually a halide ion.

Based on structural variations, over 700 anthocyanin compounds have been identified from different plant sources, and categorized under different compound types. Anthocyanidin, the aglycone molecular framework constituting the anthocyanins, have been identified based on their 27 distinct types, which produce the different anthocyanins by virtue of their structural variations. Interestingly, the anthocyanins are rarely encountered as aglycone in nature, and nearly all of them exist as glycosides, of which nearly half are acylated in their structures. The chemistry of anthocyanin colors’ involve the pH-based modulations [46]. The quinonoid base (pH 8–10) changes to pH < 2 of the flavylium cation with red to orange color which upon hydration produces the carbinol pseudo-base between pH range 3–6 with a colorless hue, and then the chalcone pseudo-base is produced which also remains colorless (Figure 2). However, the color and stability of these products are also controlled by the presence of other anti-oxidants, oxygen, moisture, enzymes, metals, light exposure, and temperature, including the factors of pH and the structure of anthocyanins, of which the presence of hydroxyls and methoxy groups lowers the stability of the anthocyanins [47]. Moreover, the tannin-anthocyanin co-conjugates increases the color stability at the lower pH [48]. Moreover, the co-pigmentation of the anthocyanin aglycone with flavonoids, facilitated by metallic ions presence, also stabilizes the color. The acetylation and glycosylation of the anthocyanidins immensely contribute to the color up-keep of the glycosylated products, the anthocyanins [49]. The anthocyanins are highly sensitive compounds, and their color can also be flocculated by the presence of other compounds, such as proteins, phenolic acids, and enzymes [41,50]. However, at very high pH, the anthocyanin compounds lose their color due to their degradation [51].

## 5. Anthocyanins Extraction, Purification, and Structure Determinations

Total anthocyanidins are conventionally extracted in polar organic solvents. Acetone and their mixture with water, also, usually together, with some acid contents are used to keep the ionization state of the flavylium forms intact. A mixture of ethanol, or methanol, and water (70 to 95%, and 30 to 5%), together with hydrochloric, formic, citric, and other organic acids have been employed [52,53,54]. Lipophilic organic solvents under ultra-sonication, and slightly elevated temperature than the RT have also been utilized for seeds anthocyanins extraction [53,55]. Natural deep eutectic solvents (NADES) were used since they are biocompatible in nature, green, environment-friendly, recyclable, and sustainable. They have also been proven to be *at par* with the organic-solvents-aqueous-acid extraction media [56,57,58,59]. The methodology needed to remove the chlorophyll, and process the extraction soup for the anthocyanins rich portion, which was carried out by chromatographic means utilizing several stationary phase materials, e.g., reverse phase silica C_18_, and Sephadex^®^ to fractionate the material based on molecular weight/molecular size [60,61]. Normal silica, the SiO_2_ gel, cation exchange material, Amberlite^®^ IRC 80, Amberlite^®^ XAD-7HP, and DOWEX^®^ 50WX8 resins have also been used for the purpose [62]. A high-speed counter-current chromatography (CCC) run following the resin XAD-7^®^ treatment of the crude/semi-processed extract was also employed. Solvent system comprising *n*-butanol, ethyl acetate, and 0.5% acetic acid in 3:1:4 ratio, *n*-butanol, *tert*-butyl methyl ether, acetonitrile in ratios of 6:5:2:1 with 0.2% trifluoro acetic acid were also demonstrated to be the best solvent mixture, as also confirmed by the HPLC analysis of the obtained anthocyanin products [63,64,65].

The extraction of anthocyanin products of the grape marc were obtained from acetone-water in different ratios of the acetone (5,7, and 10) at varying temperatures (20 and 60 °C) through the high-pressure CPF (concentrated powder form) technique to yield the anthocyanins extracts in powder form. The starch and silica were used as carrier materials, and colorimetric analysis was performed to check the extraction products quality. The storage of thus extracted materials provided stable color as compared to the non-CPF extracted material containing the anthocyanins for a longer period of time [66].

In a more simplified extraction method, avoiding the methanol as the toxic entrant, water and ethanol based extractions were considered as green solvent, and were used in conjunction with UV-VIS-based spectrophotometric contents determination at different pH values [67]. The difference in the λ_max_ absorption values at pH 1 and pH 4.5 in the visible range of the UV-VIS absorption range, provided an accurate estimation of the total monomeric anthocyanins in presence of other colored materials, conjugates, and polymeric entities in the diluted extract. The cyanidin-3-O-β-D-glucoside was used as an equivalent for unknown samples [68]. An estimation of the degraded and polymerized anthocyanin-based products contributed to the color intensity, and the bisulfite based reaction was employed for estimating their contribution toward color, and ensured the monomeric anthocyanin contents, which reacted with the bisulfite reagent, thereby producing the sulfonic acid adducts that did not contribute to the anthocyanin-based color, and helped in isolating the anthocyanin based fraction [69,70,71].

Critical extraction with acidified water (0.01% HCl, pH~2.3) at 110–160 °C under 40 bars of pressure had been used, and has proved to be a highly efficient procedure [72]. Anthocyanins’ structure stabilization by sulfur dioxide with high diffusion coefficient had increased the anthocyanins solubility with water [73], and the crude mixture was subjected to various chromatographic separations including, preparative TLC, normal silica gel, cellulose, and RP column chromatography, vacuum liquid chromatography, Sephadex, CCC, ion-pair and resins-based chromatography, HPLC and UPLC analyses at laboratory and extended scales, of which the HPLC and UPLC were also used for the known contents’ quantification. The use of GC has also been recommended [74,75,76].

The purified anthocyanins structures elucidations have been achieved using various spectro-analytical techniques. Ultra-violet (UV) spectrophotometry, infrared (IR) spectroscopy, nuclear magnetic resonance (NMR) spectroscopy, high-resolution (HR), and tandem mass spectrometry as well as X-ray diffractions (XRD) have been utilized in structure determinations. The HR-MS, and tandem mass methods have been pressed to identify the specific fragmentation pathways [77,78]. The aglycones, anthocyanidins, have been identified after the sugar loss in mass fragmentations, and also differentiated the structural types by the MS/MS fragmentations observations. The cross-ring cleavages, specifically for the ring-C of the anthocyanidin structures, produced different oxonium fragments ions. The mass fragments produced by the other structure parts, the structures of the ring A and B also helped to distinguish the substitution patterns and the substitution groups [79], while the NMR protons pattern accounted for the substitution pattern/designs of the anthocyanins/anthocyanidins rings. Functional groups differentiation for 1072 cm^−1^ bending vibration for C-O-C groups for the ethereal C-O-C bond of the aglycone-sugar attachments were specific for the anthocyanin structures [80]. The NMR-based identification of the known and unknown anthocyanins with the diagnostic peaks for H-4 at downfield shift at 8.6–9.1 ppm as a singlet signal identifying the flavylium salt presence was conclusive evidence [41,81]. The sugar signals, anomeric protons, β, or of α configurations of the linked glycoside(s) provided the structure of the common molecular framework-based anthocyanin framework. A 2D-NMR spectral analyses involving homo and hetero-COR (correlation spectroscopy) NMR experiments, e.g., HMBC, HSQC, TOCSY, NOESY/ROESY had been used to identify the structurally complex anthocyanin structures with certainty [82].

## 6. Anthocyanins Quality, and Contents’ Control through Spectro-Analytical and Chromatographic Techniques

The anthocyanin products chromatographic analyses are nearly dependent on the HPLC, and the ultra-performance liquid chromatography (UHPLC) technique coupled with UV-VIS, MS, and the DAD (diode array detector) [77]. The technique have been repeatedly used for anthocyanins fingerprinting of the anthocyanins-rich plant extract for a number of plant species [83,84,85,86,87]. Use of the reverse phase columns, specifically C_18_ silica stationary phase has been frequently employed. The HPLC separations on 5 µM particle size columns have also provided desired selectivity and resolution of the anthocyanins mixtures and the mixture of anthocyanidins [67,88]. The UHPLC analysis have been found more reliable [78], and quantification of several anthocyanins in the mixture have been simultaneously achieved in precise manner. The availability of analytical standards and use of RP-HPLC has advanced the technique-based precise separations, and yields quantification of the products, and their purification. It has also contributed to their stability status by identification, purification, and proper storage. The computing methods, such as principal component analysis (PCA), has also been utilized to analyze the HPLC/LC-MS-based identification of the anthocyanins among different species of the plants rich in anthocyanins, e.g., grapes [83] and bilberry [84]. The impurity profiling of the degraded, unstable materials have been looked through. The use of acid contents as part of the solvent system of the isocratic and gradient mobile phase has also facilitated separations. However, the discovery of new structural variants, especially the sugar variations has challenged the analytical chemists. Nonetheless, the mass-based techniques have provided added advantage of the MS/MS resolutions and structure confirmations through it [53,55,77,89]. The single ion recognition and single ion monitoring have equipped the technique to visualize the co-eluting, through the mass, the generated ions into second dimension, and quantify the mass peak(s) in the chromatographic analysis. The tandem mass spectrometry (MSn) in conjunction with HPLC and UHPLC has achieved to analyze various different mass fragmentations toward identifying the unknown anthocyanin product. This also helped to compare the obtained mass fragments with the mass spectral data banks.

Anthocyanins extraction and quantification of the products are performed for commercial products’ quality, crop estimations, and storage purposes [90]. Continuous efforts to improve the extraction and isolation techniques, and use of better identification methods have pushed toward the development of rapid and comprehensive methodologies for these purposes [91,92,93,94]. Calorimetric determinations [95], though in a limited role, acidification of the polar solvents, and water-based extraction have been reported with increased extraction capacity for the anthocyanins with increased stability of the anthocyanin constituents in the anthocyanin-rich extracts [96,97]. Although, the acidified solvents also run the risk of producing the flavonols and proanthocyanidin, nonetheless, the acidified solvents are still in use [98,99,100,101]. The acidic conditions affect the stability of the anthocyanins-rich products, and they also affect the colors of the anthocyanin constituents, as well as alter the nutritional value of the product, nonetheless they are still practiced [102]. Anthocyanins, occurring in nature with a bright-red color, exist as ozone salts, while at the neutral pH, these products assume the quinoidal structure with a purple color, and the color changes to blue in the alkaline media [103,104]. The resonant structure of the flavylium ion was established to be the cause of color change by Pauling in 1939, and the pH was suggested to be playing a role [44]. The anthocyanins’ color is among the main quality factors crucial for a product’s commercial acceptance [105].

In a report on improving the yields and quality of the anthocyanins from *Zea mays*, an extraction with 70% aqueous-acetone was utilized, which at 50 °C achieved highest yields with re-extraction with time extension from 20 to 60 min. The pH differential, Folin-Ciocalteu, protein precipitation, use of protease, and BCA (bicinchoninic acid assay) methods were used for confirmation of the products and its mixture. The HPLC-PDA, and SDS-PAGE analyses were performed [106]. Use of microwave energy (469 MW) with design input predictions utilizing Box–Behnken design (BBD) of response surface methodology (RSM) to extract blackberry anthocyanins was reported [107]. Yet another report published the microwave extraction of anthocyanins from Italian blueberry [108]. A number of other reports are also available [109,110,111] from different sources, including Borage plant, which utilized choline chloride and glycerol (CHGLY)-based natural deep eutectic solvent (NADES) extraction for the optimized extraction of the anthocyanins. Effects of blanching on extraction and stability of the anthocyanins from blueberry peel was also reported [112]. The use of ultrasonic energy for intensification and yields improvement of the anthocyanins’ extraction were described by Ravanfar et al. [113]. Anthocyanins extraction based on enzymatic maceration from the grape skin is also described as an environment-friendly technique [114]. In another report, effects of co-pigmentation on stability and extraction of anthocyanins has been described which suggested the use of certain organic acids to provide the stability of the anthocyanins-co-pigmented with organic acids, to be stable for about 2 months at 10 °C storage [115].

## 7. Anthocyanins Biosynthesis, Modulation of the Enzymatic Synthesis, and Biotechnical Approach to Increase Anthocyanins Yields 

Anthocyanins biogenesis takes place as part of the late step of the flavonoids’ natural production, which starts with the formation of chalcone from the 4-coumaroyl-CoA, and 3-malonyl-CoA, through chalcone synthase enzyme [116]. The chalcone is subsequently converted to naringenin as a first three-ring based flavonoid structure. The hydroxylation, and reduction processes takes place to convert the naringenin-based flavonoid moiety to anthocyanidins using dihydroflavonol reductase, and anthocyanin synthase, which later forms the anthocyanins through glycosylation by the desired sugar molecule [116].

The principal route to biogenesis of anthocyanins branches out from the core flavonoid pathway, and is demarcated by the dihydroflavonol 4-reductase (DFR), and leucoanthocyanidin dioxygenase (LDOX) [117]. The starting amino acid, phenylalanine (Phe), is transformed into cinnamic acid by phenyl amine ammonialyase (PAL), and sequentially into coumaric acid by cinnamate-4-hydroxylase (C4H), which is converted to 4-coumaroil CoA by the 4-coumaroil-CoA ligase (4CL). The 4-coumaroil-CoA is condensed with malonyl-CoA, and produces naringenin chalcone by the participation of chalcone synthase (CHS), which is transformed by the chalcone isomerase (CHI) into naringenin, and dihydrokaempferol, a dihydroflavonol product, and to the dihydroquercetin from the flavanone 3-hydroxylase (F3H), as well the flavonoid-30-hydroxylase (F30H), respectively. The sequence ultimately leads to the formations of leucocyanidins, cyanidins, and anthocyanins by dihydroflavonol reductase (DFR), anthocyanidin synthase (ANS), and UDP-glucosyl-flavonoid-3-O-glycosyltransferase (UFGT), respectively. The anthocyanins production occurs in the cytosol, and the products are stored in the cellular vacuoles [118,119]. The involvement of genes, signaling pathways, and the activators and repressors regulators of the anthocyanins production have been identified in *Arabidopsis thaliana,* and maize based experimentations with the involved biosynthetic details [120,121].

Anthocyanins enzymatic synthesis regulation and yields increment are achieved through the regulation of the gene expression involving the biogenic pathway; the regulation can be at transcriptional, post-transcriptional, post-translational, and epigenetic levels [122,123]. At the genetic levels, the tri-methylation interactions between the lysine-4 (Lys^4^) on histone H3 (H3K4me3) and chromatin, SWR1, are crucial for anthocyanins biosynthesis. In observance of the *Arabidopsis thaliana,* the histone H2 (H2A.Z) variant downregulated the accumulation of anthocyanins through repression of the expressions of the involved genes, and both the histones were found to be antagonistic in actions to each other. In mutants with increase in H3K4me3, an increase in anthocyanins amount was suggested. The expression of genes regulated by R2R3-MYB transcriptional factors, e.g., MYB11, MYB12, and MYB111 [124], and by the ternary complex, MBW complex, formed from R2R3-MYB, bHLH, and WD40 factors in the transcriptional levels, were controlled for the anthocyanin biosynthesis. MYB75, the transcription factor belonging to R2R3-MYB family was also found to be part of the MBW complex [125], and the plant (*Arabidopsis thaliana*) overexpressing the MYB75 gene exhibited higher accumulations of the anthocyanin compounds, whereas the myb75 mutants showed lower levels of anthocyanins [126,127], which has been shown in *Arabidopsis thaliana* and the *Actinidia chinensis*. For *Arabidopsis* 35S::AcMYB75 plants, the altered expression of the biosynthetic genes have been reported to produce significant levels of anthocyanins in the leaves which also involved the ternary complex-transcription factors MYB90, MYB113, and MYB114 of the family R2R3-MYB [128]. The ternary complex factors of the bHLH family, which included GLABRA3 (GL3), TRANSPARENT TESTA 8 (TT8), and ENHANCER OF GLABRA3 (EGL3) play specific roles in response to environmental stimuli [129], while the transcription factors of the WD-40 family, the TRANSPARENT TESTA GLABRA 1 (TTG1) was found involved in regulating the anthocyanin biosynthesis. The TTG1 encodes a protein consisting of 341 amino acids with four WD-40 repeats, and is not responsive to external stimuli [130,131], while the ttg1 mutant alters the anthocyanins production [132,133]. However, the presence of repressors inhibits the involved genes transcription, also with link to the promoters, such as, MYB7 and MYB4 of the R2R3-MYB family, and inhibits the anthocyanins synthesis. This involved the inhibition of ternary complex formation factors, e.g., CAPRICE (CPC), and MYBL2 of the R3-MYB family [134,135]. The transcriptional factors, AtMYB7 and AtMYB4, through the repression of DFR and UGT genes, also inhibited the anthocyanins synthesis. The mutants, atmyb7 and atmyb4 increased the expression of the involved genes, and produced more anthocyanins [134]. However, the overexpression of CPC gene in rivalry to the transcription factors, MYB75 and MYB90, exhibited altered effects on the synthesis of anthocyanins [135]. The post-transcriptional, and post-translational mechanisms are less understood, but several microRNAs, at post-transcriptional level, regulated the expression of MYBL2, SPL, and MYB75 factors, and adversely affected the anthocyanin biosynthesis. The microRNA, miR858a involved in repressing the MYBL2 leads to anthocyanins biosynthetic pathway activation [136]. The miR828 targeted the MYB75, MYB90, and MYB113 factors, which were strongly expressed in *Arabidopsis thaliana,* and provided reduced levels of PAL, CHS, CHI, F3H, F3′H, DFR genes, resulting in decreased productions of anthocyanins [137]. The exposure to light was also found to affect the anthocyanin compounds in the plants. The activity of the MYB75 factor through MAP-KINASE-4 (MPK4), and its degradation through proteasome [138] affected the anthocyanins biosynthesis. Under light exposure, the MYB75, phosphorylated by MPK4 increased the stability of the gene and its activity, thereby resulting in excess anthocyanins production in the cells cytosol, while in the dark conditions, the MYB75, and the MYB90 factors were found to be degraded through proteasome by the COP1-SPA-105 (CONSTITUTIVELY PHOTOMORPHOGENIC1/SUPPRESSOR OF PHYA-105) complex [139]. Nevertheless, the cop1 and spa mutants helped to produce more anthocyanins in a 24 hr cycle in both dark and light situations. Moreover, the water conditions also induced anthocyanin production by the cells, but the mechanism of which is still not clear. Under drought conditions, the miR156 regulation, through involvement of increased levels of the plant hormone, abscisic acid, responded to increased accumulation of anthocyanins [140]. A moderate miR156 microRNA overexpression suppressed the SPL13 gene, and overexpressed the WD-40-1 and DFR family genes to slightly overproduce the anthocyanin products [141]. The plants’ response to light and UV-B rays’ duress also positively affected the anthocyanins production [142].

Classical genetic approaches and state-of-the-art genetic engineering techniques have been adopted for increased anthocyanins production in plants. Conventional breeding through wild and commercial varieties resulting in specific cultivars also produced higher contents of anthocyanins. The dominant and recessive alleles, Aft and atv, respectively, hosted into *Solanum lycopersicum* (tomato) from *Solanum chilense* and *Solanum cheesmaniae*, produced the Indigo Rose cultivar which produced higher contents of the anthocyanins which were mostly confined to the outer skin layers of the fruit [143,144].

The transcription factors, Delia (Del) and Rosea 1 (Ros1) from snapdragon were introduced into tomato [145], and intense anthocyanic purple colors were obtained. However, the tomato varieties holding the non-functional SIAN2-like alle were incapable of producing anthocyanins [42].

Nonetheless, the extensive needs, and wider-angle applications of the plant anthocyanins have continuously driven the refinement of the recombinant means to produce anthocyanin class of compounds.

## 8. Anthocyanins’ Synthesis and Semisynthetic Routes to Approach Newer Analogs

Anthocyanins are considered an important group of flavonoids due to their common structural features, and for providing medicinal and nutritional benefits. In this context, the isolation and characterization of anthocyanins from the natural sources, as well as development of their semi-synthetic and synthetic protocols have attracted the researches, and race to synthetic methods development is going on. In an approach toward the total synthesis of anthocyanidin, a chalcone intermediate, 2-hydroxychalcone, obtained through Heck reaction, utilizing the condensation of 1-(3,4-dimethoxyphenyl)-prop-2-en-1-one, and O-acetoxy-iodo-benzene was achieved [146]. Another approach utilized the hydride abstraction strategy to prepare the anthocyanidins, and isoflavylium salts from the benzopyrans in good yields [147]. The total synthesis of apigeninidin, luteolinidin, and 5, 7-dihydroxyflavylium chloride were achieved through single step preparation from an acetylated derivative. The condensation of 2,4,6-triacetoxybenzaldehyde and acetophenone derivatives in anhydrous methanolic-HCl yielded 3-deoxyanthocyanidins in high yields [148]. Conversion of rutin to cyanidin-3-O-rutinoside has been reported by Oyama et al. using Zn-based reduction reaction, and aerial oxidation [149]. Synthesis of red-wine metabolites, malvidin-3-O-β-D-Glucuronide is also available [150]. The naturally encountered major anthocyanin, cyanidin-3-O-glucoside was prepared as a tracer compound labelled with ^13^C. Diethyl [2-^13^C]-malonate and [1,3-^13^C(2)]acetone were used to produce penta-^13^C(5)-labelled anthocyanin, [6,8,10,3′,5′-^13^C(5)]cyanidin-3-O-β-glucoside chloride [151]. These synthetic strategies have the potential to produce various different molecular framework of newer anthocyanins based on the feasibilities of the substitutions in their respective synthons, intermediates, and the couplers involved, as part of the condensation structures. 

A number of patents (filed and granted) have addressed the issue of anthocyanin compounds synthesis. The US patent, US8513395B2, disclosed the use of sugar moiety coupling to a substructure which was coupled to the (western-side) half synthetic intermediate to provide the anthocyanin [152].

Another USP application, US20090111975A1, similar to US8513395B2, protected the same synthetic route devised by the assignee to achieve anthocyanin structure. Two of the European patents, EP0348121B1 and EP1891086B1, disclosed the methodology for the synthesis of anthocyanidins, involved intermediate, and the anthocyanidins preparation from the sugar coupling with a synthon as the intermediate product, which was coupled to the available second synthon to provide the final anthocyanin product, as contained in the European patents, EP0348121B1 and EP1891086B1 [152]. These applications are in parallel to the USP 8513395B2, as discussed earlier. The world patent, WO2006134352A1, also disclosed the same methodology in parallel, world-wide protected the intellectual property of the authors [152].

**Figure 3:**
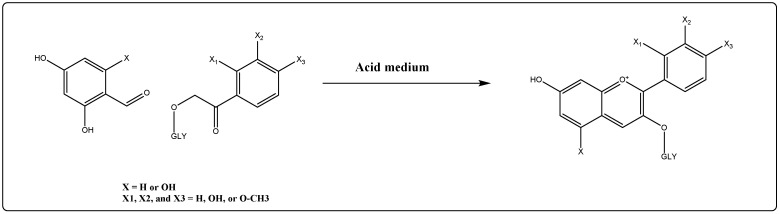
General synthetic route for preparation of anthocyanins.

The research group of Him et al. synthesized 4′-hydroxy flavylium ion chloride salts from salisaldehyde, and *p*-hydroxy acetophenone condensation. The two anthocyanidins were prepared to evaluate the antioxidant potentials of the anthocyanidins containing 5,7-di-hydroxy groups. It was compared for anti-oxidant potential levels of the 4-OH derivatives of the synthesized anthocyanidins [153], wherein most of the anthocyanidins generally had the free hydroxyl moieties at the C-5, C-7, and C-4′ positions. Moreover, a complete chemical synthesis of cyanidin-4′-O-methyl-3-β-D-glucoside had been formerly conducted by the group of Cruz et al. [154] through Robinson’s acidic aldol condensation between 2,4-diacetoxy-6-hydroxybenzaldehyde and 2-(2,3,4,6-tetra-O-acetyl-β-D-glucopyranosyloxy)-3′-benzyloxy-4′-methoxy acetophenone. Similarly, Barcena et al. have synthesized three different anthocyanidins through condensation of 2,4-dihydroxy benzaldehyde with three different acetophenone derivatives in presence of acetic acid and sulfuric acid to prepare 7-hydroxy-2-(2-methoxyphenyl)-chromenylium hydrogen sulfate, 7-hydroxy-2-(3-methoxyphenyl)-chromenylium hydrogen sulfate, and 7-hydroxy-2-(4-methoxyphenyl)-chromenylium hydrogen sulfate [155]. It has been found that the 2′-OCH_3_ and 4′-OCH_3_ substituted anthocyanidins exhibited higher antioxidant activities as compared to the 3′-OCH_3_ in FRAP (Ferric ion reducing antioxidant power) based antioxidant assays, and that can be attributed to both the resonance and the inductive effects of the compounds [155]. A literature overview revealed that the mixture of phenolic aldehydes, e.g., salisaldehyde, together with the acetophenone derivatives in acidic media were the common synthetic pathway used to synthesize anthocyanidin derivatives which were further glycosylated to prepare the designated anthocyanins [155] (Figure 3).

The most abundant, and the strong antioxidant, as well as possessing superior hepatoprotective activity, the anthocyanin, cyanidin-3-O-β-glucoside [156] was synthesized from (+)-catechin glucoside through the flav-3-en-3-ol intermediate [157]. Cyanidin-3-O-β-glucoside has also been biosynthesized from (+)-catechin by the enzymes, anthocyanidin synthase (PhANS), and 3-O-glycosyltransferase through using the *E. coli* BL21 by participation of the combinatorial promoters directing the metabolic flux toward the UDP (uridine diphosphate)-D-glucose [158].

## 9. Herbal Medicines Traditional Uses, Toxicity, Liver Disorders, and Anthocyanins

The use of plant-based products is from antiquity, and plants have entered the traditional system of medicine as well as in the folklores of various civilizations and cultures around the world. Moreover, the old-age knowledge about the toxicity of the herbal products is also obscure, and limited. Certain plants products, their derived nutraceuticals, and food supplements, as well as dietary supplements extracts and powders have been found detrimental to health, damaging the liver and disrupting its functioning. The use of ma-huang, germander, valerian, mistletoe, skullcap, chaparral, comfrey, kava, pennyroyal oil, and excessive intake of vitamin-A are among these products [159,160]. There are also reports of excessive iron, potassium, calcium, vitamins C, niacin, folate, green tea, ginseng, black cohosh, and anabolic steroids causing the liver damage [161,162]. Certain antidepressants, antibiotics, anti-epileptics, synthetic hormones, antifungal and anti-microbial drugs [163,164,165] are also reported to cause liver damage upon their excessive uses. Indirect implications of the methotrexate, valproic acid, tamoxifen, estrogen, diltiazem, and antiretroviral drugs have been implicated in liver disorders, especially the non-alcoholic fatty liver disease (NFLD) and the liver tissue damage [166,167].

There are several liver diseases, and multiple types of liver-based malfunctioning, that are hard to diagnose, hence an early diagnosis is recommended by physicians. Nonetheless, the liver diseases can also be inherited, i.e., hemochromatosis, Wilson’s disease (copper storage in liver), and α-1 antitrypsin deficiency. The hyperoxaluria, a condition when urine contains high levels of urea as a consequence of liver making excess oxalate, owing to certain genetic mutation, and which leads to kidney failure, together with excessive oxalate accumulations in several organs. The other condition, hemochromatosis, manifests itself when excess iron is stored-up from the food, and the excessive iron is accumulated in liver, including heart, and other organs, and which leads to liver disorders, as well as cardiovascular conditions and diabetes [168]. There are several contributing factors to liver diseases, and malfunctioning, including fat accumulation (5–10%) in non-alcoholic liver, called nonalcoholic fatty liver disease (NAFLD), parasitic and viral infections, i.e., hepatitis A, B, and C, excessive weight gain, and permanent obesity, excessive alcohol abuse by individuals, different drugs’ abuse and their adverse reactions, exposure to toxins, certain harmful herbal products, and the immune attacks led liver disorders, i.e., auto-immune hepatitis, primary biliary cholangitis, primary sclerosant cholangitis, and type-2 diabetes, as well as malignancies causing liver tissue and bile duct cancers, and the liver adenoma [169,170]. Some persisting adverse conditions, malfunctioning, and infections, including chronic inflammation can also lead to liver cirrhosis, a life-threatening situation, which can be controlled, also due to self-regeneration capacity of the liver tissue. However, the warning signs of looming liver disorders include jaundice (yellow coloration of eyes and skin), abdominal pain and swelling, darker urine, pale stool, nausea, chronic fatigue, loss of appetite, and itchy skin, etc. Most of the liver diseases can be confirmed by blood tests, scanning CT (computed tomography), and MRI (magnetic resonance imaging), ultrasound, and the biopsy. However, the treatment for liver disorders depends on the diagnosis and the disease condition. Life-style modifications of removal of alcohol consumption, weight loss, control of diabetic conditions, removal of processed carbohydrates, red-meat, *trans*-fat, and high-fructose corn syrup from the diets, light exercise (30 min/day) have also been recommended to help [171]. Among short-term liver disorders, the acute liver failure, where liver functioning is severely affected, or stopped within days, or weeks, and which is caused by overdose of prescription and OTC drugs, acetaminophen overdose, as well as severe infection, or chemicals led damage, i.e., cyclophosphamide, acrylamide, endotoxin, d-galactosamine, palmitic acid, and carbon tetra chloride, are known. Among the herbal products, used traditionally for a long time for liver disorders include *Phyllanthus niruii*, *Silybum marianum* (milk thistle), *Glycyrrhiza glabra* (licorice root extract, and glycyrrhizin), and berry-based products [172]. The use of carom seeds, papaya, cumin seeds, garlic, and carrot is also recommended as part of the traditional plants-based products for liver therapy [173]. In this context, the colored plants have been used by humans in different aspect, i.e., foods, medicines, to enhance the mood, as well as remove the environmental stress. The anthocyanins, therefore, have been used as counterpart of the human diets long back, and have been utilized in the ancient traditional applications in treatment of various diseases (Table 1). For instance, anthocyanins-rich plant parts, e.g., berries, fruits, seeds, and leaves, have been used by the North American Red Indians, Europeans, and the Chinese as part of their traditional herbal medicines to cure and prevent several other diseases, though at times, included their use in liver disorders [174]. 

**Figure 4:**
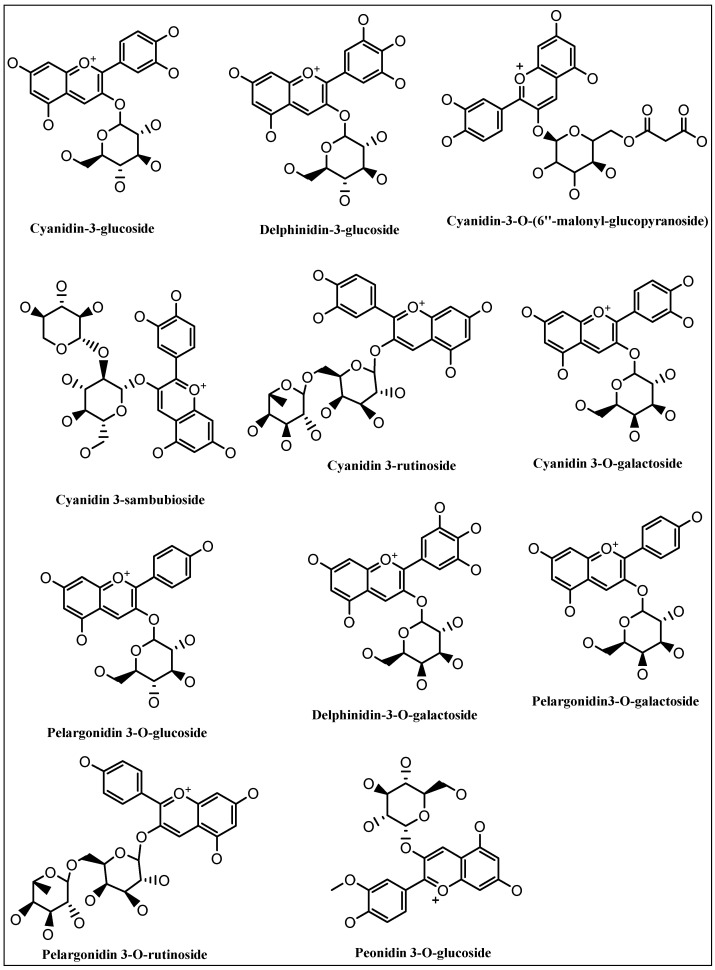
Common anthocyanins present in plants used in the treatment of liver disorders.

As the current concern deals with the traditional uses of various anthocyanins-rich plants, the Table 1 provides examples of the uses of the anthocyanins rich plants for prevention and treatment of various disorders. The major anthocyanins’ structures listed in Table 1 are presented in Figure 4.

## 10. Anthocyanins’ Metabolism in Liver

**Figure 5:**
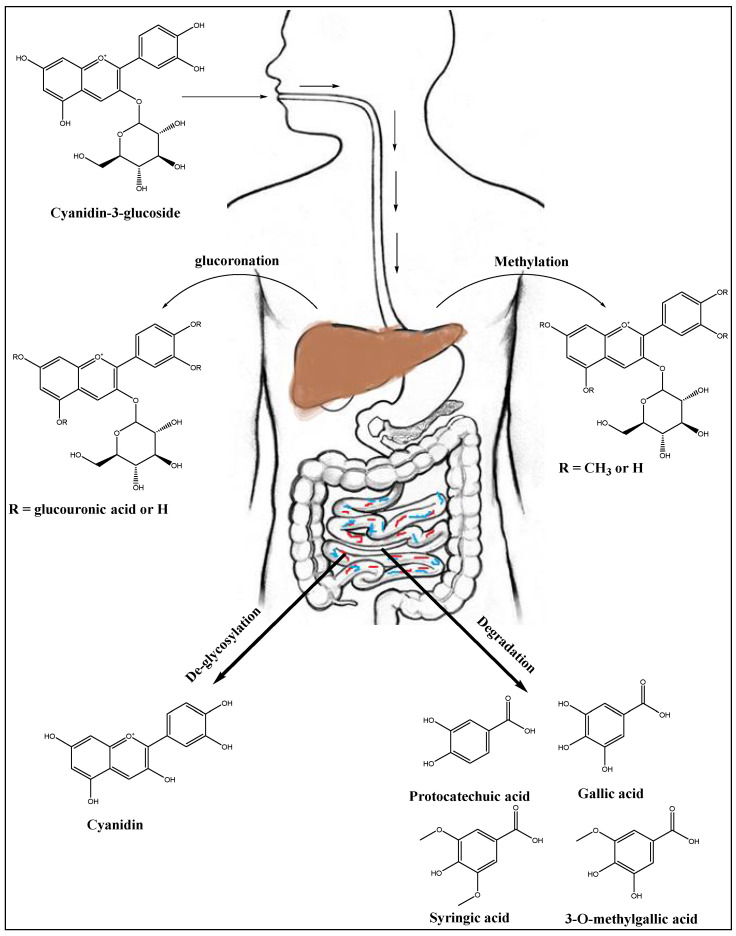
Proposed metabolic products of anthocyanins in humans.

Following the anthocyanins consumption through oral route, the absorption is followed by the stomach, and the gastrointestinal tract (GIT), where the distal lower region absorbs the majority of the products and the metabolism of the product takes place. The anthocyanins undergo extensive microbial transformation and lead to phase II metabolism in humans. The microbial–human hybrid metabolites also passed through the GIT lumen, and increase the already lowered bioavailability, and its subsequent metabolic products presence in the systemic circulation [42]. These metabolites include phenolic acid, phenolic-conjugate products, hippuric acid, phenyl acetic acid, and phenyl propionic acid, as obtained from the major anthocyanin product, cyanidin-3-O-β-glucoside, from the anthocyanins mixtures. However, the delphinidin-3-O-rutinoside, cyanidin-3-O-rutinoside, delphinidin-3-O-glucoside from blackcurrant are directly absorbed in their molecular form, and are excreted through urine as the intact glycosylated with other metabolites [69]. The anthocyanins outreach to the liver is followed through systemic supply, and according to the observations by several research groups [184,185,186,187], the anthocyanins could be considered as liver-protecting agents, with specific mechanism, and their high antioxidant potential. However, there is an important question that needs to be answered about the anthocyanins’ safety and efficacy as well, in detail. This question includes the structure(s) and nature of the anthocyanins metabolites in liver, and what is their safety/toxicity status? As a part of the answer to this question, Curtis et al., conducted a randomized, placebo-controlled trial to evaluate the safety of chronic consumption of anthocyanins on the heart, liver, and kidney biomarkers in 52 healthy postmenopausal women volunteers [188]. The study established the safety of chronic consumption of anthocyanins-rich plants, as the liver, kidney, and heart’s functions biomarkers were measured, and were found within the acceptable range after 12 weeks of chronic consumption of elderberry extract [188]. The study highlighted the safety of the dietary anthocyanins for post-menopausal women without any added cardio-protective benefits of the berry. Additionally, the anthocyanins accumulation, and degradations have been investigated in different animal-models; for instance, the accumulation of anthocyanins in pigs supplemented with blueberries for four weeks were investigated by Wilhelmina, et al. [189], and it was found that the anthocyanins were accumulated as an intact product in the liver, eyes, and brain tissues. The absorption and metabolism of the cyanidin-3-O-β-glucoside was investigated by Tsuda et al. in rats. The rats were subjected to hepatic ischemia-reperfusion as an oxidative stress model. The cyanidin-3-O-glucoside, and protocatechuic acid were detected in the plasma of the rats, however, the methylated form of the cyanidin-3-O-β-glucoside was also detected as metabolite of the cyanidin-3-O-β-glucoside in the liver, and kidneys [190]. Furthermore, the methylated and glucuronidated metabolites of the anthocyanins were also detected in the liver of rats fed with the blackberry extracts. The Figure 1 depicts the major sites of anthocyanins absorption and metabolism which were mainly absorbed from the stomach and colon [191]. The absorbed anthocyanins reach the vital organs, i.e., liver, and kidneys, through systemic circulation, where their common metabolites, methylates and glucoronates are also found [191]. Part of the anthocyanins metabolism by the gut microbiota includes de-glycosylation (conversion of cyanidin-3-O-rutinoside into cyanidin-3-O-β-glucoside, and cyanidin aglycone), and the anthocyanin products degrade to small molecules, e.g., protocatechuic acid, gallic acid, syringic acid, and 3-O-methylgallic acid (Figure 5), which supposedly contribute to reported health benefits, and biological activities of the anthocyanin molecules [192,193,194,195]. Protocatechuic acid, the main metabolite of anthocyanins [196,197], exhibits antioxidant and anti-inflammatory activities, and has been demonstrated to provide liver-protecting effects in different models of liver injury [198,199,200,201].

## 11. Anthocyanins-Based Broad-Spectrum Health Benefits

The phenolics structural basis of the anthocyanins class of flavonoid compounds lends them superior antioxidant potential with strong capacity to scavenge the physiologically produced free radicals at very low concentrations. The anthocyanins’ nature to prevent, ameliorate, and scrub the oxidative stress resulted in them exhibiting biological activities against a number of diseases and physiological malfunctioning [202], that included, besides the cancers, cardiovascular, neurological, diabetic, eye functions of vision [203], obesity, inflammation, analgesic, dysentery, and for wound-wash, which primarily can be considered probably as anti-microbial in nature, although no unambiguous support exists on this aspect till date [22,204,205,206,207,208]. The beneficial effects of anthocyanins on health has been recorded in several pre-clinical, clinical, and epidemiological studies [43,205,209,210]. However, the purified anthocyanins intake have not shown equivalent effects as the natural mixture of anthocyanins composition as part of fruits, vegetables, and food supplements [211]. The diets rich in anthocyanins, and free of anthocyanins have been prepared, and studied in animal models, and pilot levels interventional observations for comparative purposes to establish the beneficial health effects of the anthocyanins have been carried out [212]. Anthocyanins in skin care and its formulation has been reported to provide substantial advantages in skin moisture maintenance, glow, softness, and anti-aging by the pomegranate based formulation, though at experimental basis [213,214]. 

**Figure 6:**
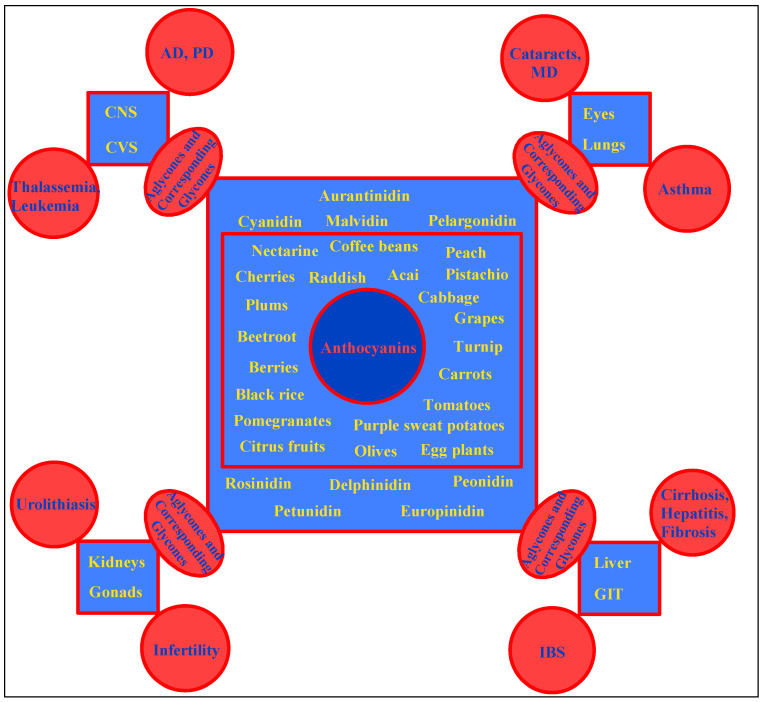
Broad spectrum biological activities of the anthocyanins.

The focus on berry fruits, socially reputed for their several health benefits, has economic significance, and industrial value as food supplements, natural food colorant, addendum to winery, and products with unique aroma [22,23]. The blueberry fruit obtained from *Vaccinium corymbosum* is a world-wide commercialized product together with the local berry produce of that particular area, Chile. The nutritional value associated with berries are considered high, and berries also have their reputation as part of superfoods [24,215]. A number of world-wide known and edible Myrtaceae berry fruits have a significant and strong reputation as potential anti-oxidant agents [25,216], and their uses in traditional Chilean and South American medicament is attributed mainly to their contents of tannins, flavonoids, and higher anthocyanins ratio [217]. For the confirmation of the anti-oxidant potentials of the anthocyanins rich extracts, which, nonetheless, is the pivotal reason of the anthocyanins’ symptomatic benefits observed in various ailments as traditional medicine, the DPPH (2,2-diphenyl-1-picrylhydrazyl), FRAP, and superoxide anion scavenging assays have been used [217,218,219]. A figurative outline explaining the major claimed and confirmed biological activities of the anthocyanins, as depicted in Figure 6, provides the names of the important plants resources containing the major structural classes of the anthocyanidin aglycones and the corresponding anthocyanins, the glycosidic derivatives, which are part of the bioactive constituents exhibiting activities in AD and PD (Alzheimer’s and Parkinson’s diseases), CNS and CVS (central nervous and cardiovascular systems) related disorders, against thalassemia, and as an anti-hyperlipidemic anti-diabetic, anti-inflammation, anti-obesity, as well as against diabetic neuropathy (DN). Anthocyanins also work against the diseases of eyes, i.e., cataracts, macular degeneration (MD), eye fatigue, glaucoma, and against diseases of the lungs, including asthma, as well as in irritable bowel syndrome (IBS), and GIT (gastro-intestinal tract) disorders, and liver diseases, including diseases of the kidneys and gonads, i.e., nephrolithiasis, urolithiasis, hyperglycemia-induced renal damage, protection against high-glucose induced renal injury in DN, and infertility have been reported. The bioactivity evaluations of mixture of anthocyanins in comparison to the specific anthocyanins are also available in plenty. [42,220,221]. 

## 12. Anthocyanin’s Dietary-Intake and Deficiency, Nutrition, and Biological Importance

The anthocyanins do not form part of the requirements of essential components of human-need nutrients, nonetheless they constitute important component of food for healthy life style, and at times customary of fruits consumption to promote better health and general well-being. An estimated 9%, 11%, and 14% of cases have been reported, where ~1.7 million fatalities were caused by heart failure, ischemic heart disease, and GIT-related cancers, respectively, and the fact have been attributed to low intake of fruits and vegetables [222]. In this context, the daily intake of flavonoids and anthocyanins have been found around 200–250 mg/day, while grape-skin based anthocyanins intake have been set at 2.5 mg/kg by the WHO-FAO. However, in the United States, anthocyanins intake has been set according to the US-FDA Nutrient Database of flavonoids at 12.5 mg/day/person, which is supported by the US-NHANES (United States National Health and Nutrition Examination Survey). Moreover, of the total intake, the cyanidin, delphinidin, and malvidin aglycones ratios were analyzed to be 45, 21, and 15%, respectively, of which the non-acylated were 77%, and acylated anthocyanins were at 23% of the anthocyanins ratio. Interestingly, no human toxicity of anthocyanins has been reported till yet, especially from the food intake, quantities of which are very infinitesimal [223,224,225,226,227]. Anthocyanins, owing to their structural features, and the linked physico-chemical properties, have been challenging to assess because of their bioavailability status in the in vivo conditions. However, studies based on blood and urine analyses have been the norm to quantitate the anthocyanins concentrations from the anthocyanins-rich foods ingestion [228,229,230]. Additionally, the anthocyanins absorbed through stomach and intestines, reach to liver and kidneys. Cyanidin-3-O-β-glucoside and pelargonidin-3-O-glucoside were known to be absorbed in their molecularly intact glucosylated form from the GIT at nearly a concentration of 100 μg/L after half an hour of their ingestion [231], and pass through liver to metabolize in the process of first-pass metabolism to produce the metabolic products which are delivered into the systemic circulation, wherein the phenolic acid predominates the other metabolites. These metabolites are considered to be of immense health benefits. However, some of the anthocyanin products reaching the intestinal canal are fast decomposed by the microbiota which are present there [232]. Notwithstanding, the contribution of gastric mucosa in shaping the bioavailability of anthocyanins is of immense importance, and is projected by the intact anthocyanins 20–25% plasma level concentrations of the total anthocyanins intake. The anthocyanins are absorbed and eliminated at higher pace than the normal flavonoids, e.g., the quercetin-based glycosides which are slow to absorb and in being eliminated. But, the anthocyanins reach faster in plasma and urine, and, at times, are not reflective of their actual concentration, either high, or at low levels of concentrations in the fruits and plants-based ingestions. The ability of the anthocyanins to cross-over the membrane, effects of stomach pH, presence of bile acids and digestive enzymes, and hydrochloric acid, as well as the food matrix present in the GIT affects their bioavailability and metabolism. Moreover, the lack of analytical instrumentations, their insensitivity to quantitatively measure, and the absence of non-invasive techniques, have made the task nearly unsurmountable. The mechanism are still under investigations, and it is not clear whether the bioavailability effects is due to the native compounds, or other structural forms arriving out of the GIT interactions results [233]. Nonetheless, there is high inter and intra-individual variations in bioavailability of the anthocyanins which is also driven by the intake response in an individual, and which seems to affect the mechanism of action, and overall bioavailability of the anthocyanins products in the systemic circulation and at cellular sites. The bioavailability variations have been attributed to food processing based on the food matrix variety, enzymes involved in anthocyanins metabolism and transport to the next and farther locations, and the intestinal and gut microbiota taking part in anthocyanins decomposition. However, there are insufficient evidence to conclusively support these notions, nonetheless, preliminary observations have been noted regarding the factors affecting bioavailability [228,234]. Among strategies to enhance the bioavailability of the anthocyanins, apart from nano and micro-encapsulations, nano-liposomal preparations, and nano-emulsions, the chemical modifications of the anthocyanin structures have been the alternate approach. The acetyl derivatizations, and to some extent, the methylation of the anthocyanins have contributed to their stability, enhanced bioavailability, and longer systemic circulation times [42,235,236,237].

The bioavailability of anthocyanins have been linked to maintenance of good health through reducing the cellular lipid peroxidation, and its strong anti-oxidant activity. Nonetheless, the anthocyanins, especially the most abundant, cyanidin-3-O-β-glucoside, has provided low bioavailability in pharmacokinetic studies [238,239], and studies have been devised to further substantiate, and increase the bioavailability of the anthocyanins through various formulation strategies, also involving nano-encapsulation [240]. Matter of the fact, recent studies have shown that the anthocyanins are more bioavailable than previously presumed. Anthocyanins mixture from red fruit were found to be non-metabolized after 30 minutes of consumption of the anthocyanins product [241]. A relative bioavailability of the anthocyanin, cyanidin-3-O-β-glucoside, stood at 12.38 ± 1.38%, and 5.37 ± 0.67% were out in urine after 48 h of oral ingestion [239]. 

Anthocyanin are also appetite stimulant, choleretic in action, and are recognized as potent nutraceuticals. The products, being bioactive component, exert several pharmacological activities, including the substantial anti-cancers, which is one of the most-searched bioactivity. The anthocyanins also reduce the inflammation, and modulate the immune system’s responses, which supposedly is considered to be a result of their antioxidant potential [242]. The cyanidin and delphinidin have been observed to inhibit epidermal growth factor receptor in cancer cells, whereas the malvidin stood at lowered effectiveness against EGF receptors [243]. Anthocyanins also neutralize the enzymes working against connective tissues, and prevent oxidative stress-led damage to connective tissue. They also tend to repair the damaged proteins’ parts in blood-vessel walls. The structural variations of the anthocyanins play pivotal role in their biological activity elicitation, and the involved health benefits vary according to the molecular framework, and substitutions on the anthocyanin compounds [244].

Studies have found that nutrition utilizing anthocyanins supplements of cherry, blueberries, and grapes, in separate experimentations, have been found to increase the oxidative defenses [245,246]. Anthocyanins supplementation in the diets of 7–10 years old children improved their cognitive functions [247,248], which have also been observed in adults [249,250], and elderly [251,252,253], although results in young adults are debated.

## 13. Anthocyanins and Liver Disorders

The liver is one of the important organs of human body with capacity to regenerate. The liver is highly sensitive to xenobiotic entities, oxidative stress, and presence of toxins. It is also well-known that the liver regenerates itself, and resizes its portions after partial hepatectomy. The activation of hepatocyte proliferation, modification of the enlarged liver mass, and correction to the apoptosis process are also known [254,255]. The role of oxidative stress in restricting the liver cells regeneration is acknowledged [256,257], together with its contributions to induction, propagation, and catapult to liver diseases related complications plausible removal have been discussed [257,258,259,260]. The reactive oxygen and nitrogen species (ROS and RNS) produced as a part of normal metabolic functions are under limits to take part in physiological functions in the body, and are considered significant as primary elements in the inflammation responses in the innate immunity mechanism [257,261]. They also have physiological roles in processing of signal transduction, and normal process of ageing and cell death. The excessive production of these critical species, ROS and RNS, particularly associated with the mitochondrial dysfunctions, are also responsible for the endogenous production of ~90% ROS through the oxidative phosphorylation type of metabolic process [262]. The excessive production of ROS is associated with initiation of lipid peroxidation, DNA damage, glycoxidation, and protein oxidations, of which all are linked to promoting of several degenerating diseases, and soft tissues injury [263].

**Figure 7:**
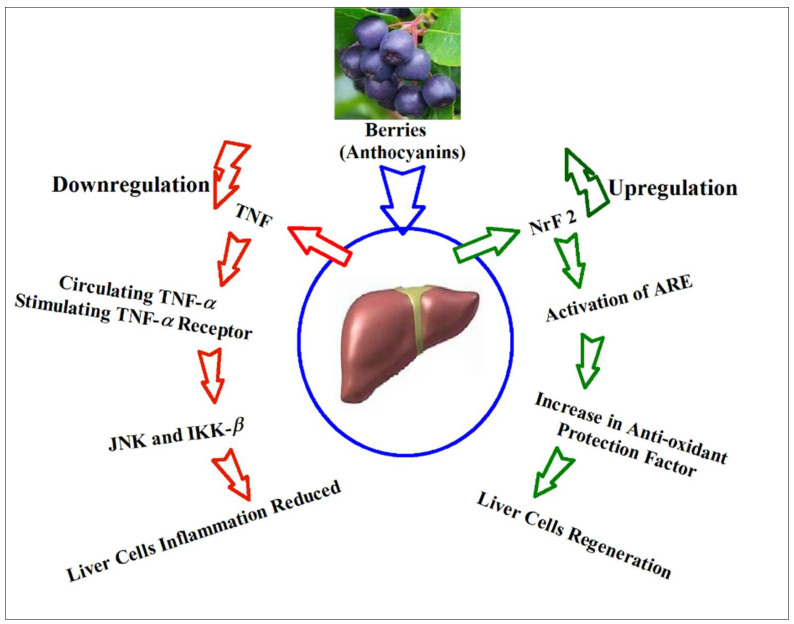
Diagrammatic representation of major hepatoprotective pathways of anthocyanins.

Among the body’s tissues, the liver is highly susceptible to aggressive injuries caused by the processes of oxidative stress [256], and the excessive production of ROS is linked to liver inflammation and fibrosis [256]. In addition, the oxidative stress is hallmark of chronic liver disease, regardless of the cause of the injury and the inducer [264]. In liver, the parenchymal cells, mitochondrion, and endoplasmic reticulum produce ROS which are primarily associated with the liver’s fatty acid oxidation activity. The largest population of resident tissue macrophages in the liver, and Kupffer cells, are highly sensitive to oxidative inducers, which derives the initiation and development of hepatic inflammation, and consequently, the fibrosis [257]. Since the body’s metabolic processes mainly occurr in the liver, and the liver cells are susceptibility to oxidative stress, there is a greater need for the presence of self-defensive mechanism in liver to scavenge ROS. The nuclear related factor 2 (Nrf2) works in the liver as a cellular redox status sensor, in which the higher levels of ROS-induced Nrf2 are released by sequestration, and translocate to the nucleus, wherein it promotes the transcription of cytoprotective antioxidant genes, as well as this activity promotes the liver cells regeneration which almost takes place through the activation of the antioxidant response element (ARE) [264]. The impairment of the Nrf2 defensive system of the liver is considered as the direct cause to increase the hepatocytes damages in response to the oxidative stress inducers, such as, toxins and high-fats diets, which are the reasons to elevate the mitochondrial production of ROS [264]. Therefore, certain agents that alleviate the reduction in Nrf2 protein levels are the promising therapeutic candidates for liver diseases treatment, and also for liver protection against oxidative stress, as well as oxidative stresses-led liver’s lipid peroxidation [265] (Figure 7).

The pro-inflammatory cytokines group, TNF, have also been established for their role in the activation of liver diseases. Therefore, the TNF inhibitors are expected to be protective agents against liver injuries, as the increased levels of circulating TNF-α stimulates the TNF-α receptors located on cells surface, and leads to activation of the stress-related protein kinases, JNK and IKKβ. The activation of JNK and IKKβ upregulates the production of inflammatory cytokines leading to subsequent liver injury as the resultant action [266,267].

The plants-based liver prophylactic, and treatment therapies are well-known in the medicinal market, and are prescribed nowadays for the treatment of liver diseases, alone, or in combination, with other drugs [172]. Certain medicinal plants have also been consumed by different societies and traditional groups as remedies for liver complaints [268,269,270]. It is also reported that some vegetables, fruits, cereals, and flowers have ability to scavenge free radicals, and protect the liver cells from oxidative stress [271,272].

## 14. Anthocyanins’ Suggestive Roles through Hepatic Biomarkers Regulation, and Biomechanistics Outlook 

The anthocyanins support of the liver is still debated, and have been opined confirmed in some recent observations. The anthocyanins in general, and cyanidin-3-O-β-glucoside in particular, reduced the ALT and AST levels in serum, as well as malondialdehyde and protein contents levels in the liver homogenate of the experimental animals [273]. The reduced levels/activities of MCP-1, IL-1β, MIP-2, collagen III, and *α*-SMA were also obtained in the rodent liver fibrosis model. The cyanidin-3-O-β-glucoside also showed strong anti-atherogenic activity [274]. The cyanidin-3-O-glucoside and other anthocyanins enhanced the cell-based AMPK activity, and ACC phosphorylation together with the carnitine palmitoyltransferase-1 (CPT-1) expression, thereby leading to increased oxidation of the fatty acids in HepG2 cells [275,276]. The attenuation of liver steatosis, and reduction of white adipose tissue messenger RNA levels of MCP-1, TNF-*α*, IL-6, and serum concentrations of TNF-*α*, IL-6, MCP-1, as well as reduction of macrophage infiltration in adipose tissue were also observed. The cyanidin-3-O-glucoside also exhibited fasting glucose levels reductions. The cyanidin-3-O-glucoside also lowered the oxidative stress through GSH (glutathione)-based antioxidant defense mechanism, and thereupon lowered the ROS production, and subsequently the hyperglycemia-induced hepatic oxidative damage. In addition to the regulation of the thermogenic and secretory functions of BAT (brown adipose tissue), it also lowered ROS production, and oxidative stress [277,278]. In a more recent report, the liver functional biomarkers’ levels were estimated after consumption of the pure anthocyanins, anthocyanin-rich extracts, and products to check the efficacy of the products and the pure compound itself, no plausible relationship were established but levels of ALT and AST were observed to be significantly reduced upon the anthocyanins intervention in healthy subjects [279]. The NAFLD has been found to be linked to type 2 diabetes mellitus, obesity, and CVS disorders. The hepatocytes accumulated triglycerides have been observed to be countered by the anthocyanins. In preliminary studies, the anthocyanins are reported to be improving the anti-oxidant and anti-inflammatory activities of the liver cells with incremental pace of the lipid, and glucose metabolisms [280]. The protective effects of the black raspberry anthocyanins against acute and sub-acute alcoholic liver disease (ALD) were investigated through observations of serum biomarkers and liver functional index biomarking parameters, which were found ameliorated, and histopathological demarcations were studied. An increased expression of NF-κB and TGF-β was found in the extracted liver. The black raspberry extract, and the cyanidin-*3*-O-rutinoside also showed cytotoxic effects on t-HSC/Cl-6, HepG2, and Hep3B, as well as induced apoptosis in HepG2 cells. The extract and the compound, cyanidin-*3*-O-rutinoside, downregulated the Bcl-2, while upregulating the Bax levels. The study also found that cytochrome-C release was increased, and caspase-9, caspase-3, and PARP in HepG2 cells were cleaved. The black raspberry, and the pure cyanidin-3-O-rutinoside exerted its protective activity through the antioxidant potential, and the apoptosis pathways [281,282]. The anthocyanins of purple sweet potatoes have also been recommended for treating the NAFAD as a supplementary remedy during prophylactic, and curative management of disease [283].

In context with the liver infections, steatosis is histological outcome of the chronic hepatitis C viral, and at times severe bacterial infections together with as an outcome of host’s metabolic risk factors involving resistance to insulin, obesity, type 2 diabetes, and hyperlipidemia. The phenomenon tends to accumulate lipids in the intracellular spaces, and it is associated with liver fibrosis, and diminished response to antiviral therapy [284]. However, simple steatosis is benign, but a synergistic combination of cellular adaptation, and oxidative damage together with the steatosis, aggravates the injury in the liver, and may lead to chronic fibrosis and hepatic carcinoma. The heightened oxidative stress, augmented vulnerability to apoptosis, and uncontrolled cells activity have been implicated in steatosis severity [285]. The current trend of tackling the liver disorders in a combined therapy for interconnected and concurrent liver ailments, e.g., steatosis, low-level hepatocellular inflammation, obesity, insulin resistance, hyperlipidemia, type 2 diabetes, hepatitis A, B, and C, haemochromatosis, and NFALD have set the goal for complementary and alternate therapeutic goals in combination with herbal adducts, especially the flavonoids and flavonoids based anthocyanins. The diseases pathology is intertwined in increased oxidative stress, inflammation, steatosis, liver injury, fibrosis, hepatic carcinoma, altered liver regeneration, and eventual cell death as the end-outcome of the stress and intervention process [286,287].

Anthocyanins-rich plants have been used in folk-medicine as remedies for several diseases including protection and treatment of liver disorders. The plants organs’ rich in anthocyanins as well as pure anthocyanin entities have also been extensively evaluated for their in vivo hepatoprotection effects against several hepatocyte oxidative stress inducers, e.g., carbon tetrachloride (CCl_4_), ethanol, acetaminophen, thioacetamide (TAA), *tert*-butyl hydro-peroxide (t-BHP), and dimethyl-nitrosamine. Certain reports have also evaluated the liver protection effects of anthocyanins-rich plants extracts using in vitro cell line models, e.g., inducing oxidative stress in HepG2 cell lines (Table 2 and Table 3). An overview provided the details of their hepatoprotective activity.

Nonetheless, the upregulation of Nrf2 protein and the down regulation of the pro-inflammatory cytokines, TNF-*α*, are two possible mechanisms adopted by the liver-protecting agents. Anthocyanins have shown potential activity in both the mechanisms. For instance, Hwang et al., observed that the anthocyanin fraction of purple sweet potato exhibited in vivo hepatoprotective effects in the *t*-BHP-induced liver injury through the intermediacy of their capacity to scavenge the free radicals, and regulate the heme oxygenase-1 (HO-1) enzyme. It was also observed that the anthocyanin fractions induced the Nrf2 nuclear translocation through activation of I3K/Akt and ERK1/2 pathways, leading to hepatocyte protection [187]. The purple sweet potato anthocyanins also exhibited liver protection against CCl_4_ and acetaminophen induced liver injuries in the mice models [290,291]. These results revealed the ability of the anthocyanins of sweet potato to attenuate the acute and chronic liver injuries. Sweet potato anthocyanins have been found to reduce CYP2E1-dependent aniline hydroxylation, and CYP2E1 protein levels, as well as FeCl_2_/ascorbate-induced lipid peroxidation, which increased the hepatocyte GSH levels due to their antioxidant power [290]. This effect of anthocyanins made them potential hepatoprotective therapeutics, especially for the possible liver injury of the acetaminophen toxic metabolite, N-acetyl *p*-benzoquinone imine (NAPQI), which in high concentration depletes the glutathione contents of the hepatocyte, and directly attacks the cellular macromolecules, e.g., DNA, proteins, and cellular lipid membranes [292]. The mulberry marc anthocyanins alleviated the liver injury induced by the CCl_4_, and markedly decreased the ALT, AST, hyaluronidase, hydroxyproline, and collagen type-III in the injured rats, and that was attributed to the antioxidant capacity of the plant [293]. The pure anthocyanin, delphinidin, has also been reported to upregulate the Nrf2, and control the expression of antioxidant protein HO-1 [288]. The compound has exhibited remarkable anti-inflammatory activity through inhibition of IL-1β-induced activation of the NF-κB, and had inhibited the expression of IKKβ, which provided the needed proof for the hepatoprotection activity of the delphinidin [294].

The effects of anthocyanins as a liver-protecting agent against several types of experimentally induced liver injuries are summarized in Table 2 and Table 3.

**Table 3:** Anthocyanins-rich fractions/extracts of plants as liver-protecting agents against experimentally induced liver injuries.

Plant’s Name	Used Extracts, and/or Pure Compounds	In Vivo/In Vitro Models and Bioactivity	Major Anthocyanins	Biomarkers, and Mode/Mechanism of Action	Refer
*Morus alba*, and species	Mulberry anthocyanins	CCl_4_ (carbon tetrachloride), in vivo model. Hepatoprotection	Cyanidin-3-O-β-glucoside	Decreased the ALT (alanine transaminase), AST (aspartate transaminase), hyaluronidase, hydroxyproline, and collagen type-III in the injured rats	[293]
*Ipomoea batatas* L.	Anthocyanins rich purple sweet potato extract	CCl_4_, in vivo model. Hepatoprotection	Peonidin-3-caffeoyl-feruloyl sophoroside-5-glucosid, peonidin 3-caffeoyl-p-hydroxy benzoyl sophoroside-5-glucoside, peonidin 3-dicaffeoyl sophoroside-5-glucoside	Reduced the AST and ALT enzymes and MDA (malondialdehyde) level; Increased the SOD (superoxide dismutase), and GSH (glutathione) levels compared to the injured CCl_4_ administered group of animals	[295]
*Oryza sativa*	Anthocyanins rich black rice bran extract	CCl_4_, in vivo model. Hepatoprotection	Cyanidin-3-O-β-glucoside, and peonidin-3-O-glucoside	Reduced aminotransferase activity in serum, enhanced SOD and glutathione peroxidase (GSH-Px) activities, thiobarbituric acid reactive substances (TBARS), and 8-hydroxy-20-deoxyguanosine levels significantly decreased as compared to the CCl_4_ intoxicated group. Liver histopathology confirmed pathological gains by ARBE administration	[186]
*Ipomoea* *batatas*	Anthocyanins rich fraction of purple sweet potato extract	In vivo, ethanol, acetaminophen, and, CCl_4_. Hepatoprotection, and treatment	3-O-(6-O-trans-caffeyt-2-O-~-glucopyranosyl/3-glucopyranoside)-5-O-glucosides of cyanidin, and peonidin	Treatments of mice with anthocyanins fraction in dose dependent manner, and reduced the CYP2E1-dependent aniline hydroxylation, and CYP2E1 protein levels. Antioxidant effects on hepatic GSH level, and GSH S-transferase activity were up-regulated in FeCl_2_/ascorbate-induced lipid peroxidation in mouse liver homogenates, also showed superoxide radical scavenging activity.	[290,291]
*Hibiscus sabdariffa* L.	Anthocyanin-rich extract	In vivo, thioacetamide (TAA)-induced hepatotoxicity. Hepatoprotection	Cyanidine, delphinidin derivatives, cyanidin-3,5-O-di-glucoside, cyanidin-3-O-sophoroside-5-glucoside	Reduced the serum levels of ALA, AST, and hepatic malondialdehyde, decreased hepatic inflammatory markers, including TNF-α, interleukin-6, and INF-γ, decreased the immuno-positivity of NF kappa-B, and CYP2E1 in liver tissues	[185]
		In vivo *tert*-BHP-induced cytotoxicity in rat			[176]
		CCl_4_ in vivo model. Hepatoprotection			[296]
*Aronia melanocarpa*	Fruit juice	CCl_4_, N-nitroso diethyl amine, Paracetamol in vivo model. Hepatoprotection	Cyanidin-3-O-galactoside, cyanidin-3-O-arabinoside, cyanidin-3-O-xyloside and cyanidin-3-O-β-glucoside	Reduced necrotic changes in rat liver and inhibited increase of plasma AST and ALT activities, MDA formation induced by CCl_4_. Increased liver GSH contents. Decreased the activities of enzymatic markers of cytochrome P450, CYP1A1 and 1A2.	[297,298,299]
*Justicia* *spicigera*	Ethyl acetate fraction	CCl_4_ in vivo model. Hepatoprotection	Peonidin 3,5-O-di-glucoside, malvidin 3,5-O-di-glucoside, and petunidin 3,5-O-di-glucoside	Improvement in liver function indices and oxidative stress markers. Increased SOD and GSH, and decreased MDA.	[184]
*Vaccinium* sp.	Berry pomace extract	In vitro hepatic cell line HepG2 proliferation. Hepatic cells protection	Procyanidin dimers	Protects hepatic cells from oxidative damage.	[300]
*Solanum* *tuberosum* L.	Purple potato’s anthocyanins rich extract	In vivo, alcoholic liver disease mouse model. Hepatoprotection	Petunidin-3-coumaroyl-rutinoside-5-glucoside, peonidin-3-coumaroyl-rutinoside-5-glucoside, petunidin-3-O-glucoside, petunidin-3-rutinoside-5-glucoside, pelphinidin-3-coumaroyl-rutinoside-5-glucoside	Higher levels of SOD and reduced GSH enzymes, reduction in formation of malondialdehyde, protected against alcohol-induced detrimental levels, maneuvered the activity of cytochrome P450 2E1 (CYP2E1)	[301]
*Ipomoea* *batatas*	Anthocyanin fraction	Dimethyl nitrosamine-induced liver injury in rats. Hepatoprotection	Cyanidin-3-O-β-glucoside chloride, malvidin-3-O-glucoside, pelargonidin-3-O-glucoside chloride, and peonidine-3-O-glucoside chloride	Induced Nrf2 mediated antioxidant enzymes, and reduced the COX-2, and iNOS expressions, reduced inflammation through NF-KB inhibition	[302]
*Hibiscus sabdariffa*	Water extract, and anthocyanins	Paracetamol-induced hepatotoxicity in rats. Hepatoprotection	Anthocyanins	Increased GSH and SOD levels, decreased ALT and AST	[303]
*Colocasia* *antiquorum*	Ethanolic extract	Paracetamol, and CCl_4_ toxicated rats. Hepatoprotection	Cyanidin-3-O-β-glucoside, pelargonidin-3-O-glucoside and cyanidin-3-O-rhamnoside	Decreased ALT, and AST levels	[304]
*Vaccinium myrtillus* and *Ribes nigrum*	Anthocyanins-rich extracts	Acetaminophen-induced hepatotoxicity in rats. Hepatoprotection	Glycosides of cyanidin, peonidin, delphinidin, petunidin, and malvidin	Normalized activities of glutamate oxaloacetate and glutamate pyruvate transaminase, prevented APAP-induced plasmatic and tissue alterations in biomarkers of oxidative stress	[305]
*Raphanus* *sativus* L. (Red radish)	Anthocyanins fraction	CCl_4_ in vivo model. Hepatoprotection	Pelargonidin derivatives	Reversed the alteration of biochemical parameters to normal	[179]
*Raphanus* *sativus* L. var. *niger*	Fermented roots	In vivo model for the methionine, and choline-deficient, diet-induced non-alcoholic fatty liver in mice. Hepatoprotection	Pelargonidin derivatives	Decreased lipids in 3T3-L1 adipocytes by downregulating adipogenic transcription factors, sterol regulatory element-binding protein 1c, CCAAT/enhancer-binding protein α, peroxisome proliferator-activated receptor γ, and lipid accumulation-related genes adipocyte protein-2, as well as fatty acid synthase. Decreased ALT, AST, TG levels. Deceased expression of iNO synthase, suppression of the inactivation of macrophages, and Kupffer cells in liver. Inhibition of α-smooth muscle actin, transforming growth factor β-1, and collagen type-I α-1 chain leading to reduced liver fibrosis.	[306]
*Raphanus* *sativus* L. var. *niger*	Aqueous extract of roots	In vitro model in HepG2 cells. Hepatoprotection	Pelargonidin derivatives	Induced quinone reductase activity, and expression of multiple phase I, II detoxification enzymes in the HepG2 human hepatoma cell line	[307]
*Malvaviscus* *arboreus* Cav	Aerial parts extracts	CCl_4_ in vivo model. Hepatoprotection	Cyanidin-3-sambubioside	EtOAc (ethyl acetate), and CH_2_Cl_2_ (Dichloromethane) extracts significantly reduced the liver injury in rats as indicated by the reduced levels of ALT, AST, ALP, TB, and MDA, comparatively the EtOAc fraction enhanced total antioxidant capacity of liver at the maximum.	[308]
*Cornus mas* L.	Anthocyanins rich fraction	Lipid peroxidation, oxidative stress in the livers of cholesterol-fed rabbits	Delphinidin 3-O-galactoside, cyanidin-3-O-galactoside, Cyanidin-3-O-robinobioside, pelargonidin-3-O-galactoside, pelargonidin-3-O-robinobioside, cyanidin, and pelargonidin.	Decreased lipid peroxidation, decreased MDA levels, and reduced oxidative stress, an increase in liver GSH found.	[309]

## 15. Anthocyanins Roles in Hepatocellular Longevity, Hepatic Carcinoma and Liver Cancer 

Anthocyanins have also been reputed with liver longevity. Kunming mice administered with D-galactose to accelerate ageing were intervened with anthocyanins administrations, and liver histology and functions were evaluated after eight weeks. Western blot analysis was used to assess the genes involved in DNA damage signaling pathways. Hepatic tissue injury, fibrosis were found reduced, while the liver functional biomarkers were found to be delayed in their levels’ reductions. The anthocyanins administrations maintained the stability of the GSH redox system (GSH-PX, T-SOD and MDA), as found in the plasma and liver, together with the reduced levels of the inflammatory factors, i.e., IL-1, IL-6, and TNF-α were observed. Expression levels of the sensors (ATM, ATR), mediators (H2AX, γ-H2AX), and effectors (Chk1, Chk2, p53, p-p53) of the DNA-damage signaling pathways were found reduced [310].

Anthocyanins role in cancer prevention and cure has been much deliberated by the researchers. Anti-cancer effects of the anthocyanins have been suggested to be connected to a number of different biological activities mediation including the anti-inflammatory, antioxidant, anti-mutagenesis, inhibition of cells proliferation through modulating the signal transduction pathways, cell cycles arrest, inhibition to induction of cells differentiation, as well as apoptosis, and autophagy of the cancer cells. The reversal of drug resistance, increased sensitivity to chemotherapeutic agents, anti-invasion and anti-metastasis have also been suggested to be involved in ameliorating the cancerous situations. A data analysis of the basic findings, in vivo and in vitro, inferences from clinical trials, as well the herbalists and traditional healers practices based information was analyzed [311]. The anti-cancer effects of anthocyanins have also been reported by Longo et al. The anthocyanins-rich extracts obtained from the Mediterranean ever-green shrubs’ berries from *Phillyrea latifolia* L., *Pistacia lentiscu* L., and *Rubia peregrina* L. were examined for their anticancer activity, and autophagy inhibition enhanced anthocyanin-induced apoptosis in hepatocellular carcinoma was observed to be working as the mechanistic aspect of the anti-cancer action. The autophagy was established through observation of up-regulation of the autophagy inducer, elF2α, and down-regulation of the autophagy inhibitors, i.e., mTOR and Bcl-2 which led to enhanced expressions of LC3-II. The autophagy was replaced with the apoptosis, also confirmed by the activation of Bax, cytochrome *C,* and caspase 3. The terminal deoxy nucleotide transferase mediated dUTP nick-end labeling–positive fragmented nuclei, and cancer cells with sub-G_1_ DNA contents that were prevented by z-VAD, confirmed the notion. The autophagy inhibition either by 3-methyladenine, or Atg5 small interfering RNA, prompted the anthocyanin-led apoptosis. Hence, intervention of autophagy inhibitors in combination with anthocyanins and anthocyanins rich extract/products can be beneficial in controlling hepatic cancer [312].

On the mechanistic front, the cytochrome P450 family enzymes CYP1, CYP2, and CYP3 played major roles in metabolism of ~75% of all administered drugs of herbal and synthetic origins, together with other chemical entities reaching the liver. The NAFLD disorder represented a noticeable reduction in these vital enzymes. In an experiment dealing with microsomes isolated from human liver samples, the microsomal CYP1A2, CYP2D6, and CYP2E1 mRNA levels were found to be decreased with the NAFLD progression, while the CYP2A6, CYP2B6, and CYP2C9 mRNA expressions were found increased. The microsomal protein expression of CYP1A2, CYP2C19, CYP2D6, CYP2E1, and CYP3A4 reduced with the progressing NAFLD. The enzymatic activity of CYP1A2 and CYP2C19 were increased with the progressing NAFLD, while the activity of CYP2A6, and CYP2C9 were found increased with NAFLD severity with different drugs metabolism. Pro-inflammatory cytokines, TNF-alpha, and IL-1beta were observed along with the decreased P450 enzymatic activity. The increased enzymatic activity of the CYP2C9 during higher degrees of NAFLD progression related with the increased hypoxia-induced factor-1alpha expression in NAFLD’s late stage [313]. The roles of CYP 450 enzymes is also more pronounced in the detoxification of the xenobiotic materials [314,315,316]. The oxidation of the heme-thiolate cysteine to a sulfenic acid (-SOH) and the heme-thiolate insensitive routes are the key step in the oxidative step involving the CYC 450 family of enzymes. This is a redox-regulated process [317]. Obesity, considered to be related to a decrease in CYP2C and CYP2E1 activities, is also regulated through the liver conditions [318]. Moreover, the CYP enzymes isoforms, with respect to genic polymorphisms, and drug metabolism have major roles in metabolism and cancer initiation. They can activate pro-carcinogens to ultimate carcinogens through exogenous substrates which constituted the majority of drugs, and other known chemical carcinogens through, primarily, in liver, but also in other organs. The clinically most relevant CYP2D6, CYP2A6, CYP2C19, CYP2C9, CYP1B1, and CYP1A2 enzymes have been in focus [319].

## 16. Anthocyanins’ Structure Types, Hepatoprotection and the Evolving SAR

The structure activity relationships (SAR) of the anthocyanins toward hepatoprotection have not been deliberated. However, the antioxidant capacity of the anthocyanins is a key indicator of the activity of these compounds as liver-protecting agents against oxidative damages are experimentally induced through carbon tetrachloride, and acetaminophen. Therefore, the SAR for the antioxidant activity of the anthocyanins could be indicative of the potential activity of the anthocyanins as a hepatoprotective agent. The anthocyanins (the glycoside from) have been found to be more stable than the corresponding anthocyanidins (the aglycone form) [320,321]. However, the antioxidant activity of the anthocyanidins has not been significantly affected by the mono-glycosylation [321]. The effects of glycosylation type and pattern (position) on the ring system of the anthocyanins affect the antioxidant activity of the anthocyanins. It has been also investigated by the group of Cyboran-Mikołajczyk et al., [322]. They recorded that the antioxidant effects of cyanidin-3-O-mono-glycosides, i.e., cyanidin-3-O-β-glucoside, cyanidin-3-O-galactoside, and cyanidin-3-O-arabinoside, were not significantly different from the aglycone, cyanidin, as they effectively protected the lipids against 2,2′–azo bis(2-amidinopropane) dihydrochloride (AAPH)-induced oxidation. However, it recorded significantly lower antioxidant potential for the cyanidin-3-O-rutinoside, and cyanidin-3-5-O-di-glucoside, which indicated the importance of the free -OH groups of the B-ring (at positions C-3′ and C-4′), and A-ring substitutions at position C-3 for the antioxidant activity of the cyanidin. These results also confirmed the effects of the number of sugar moieties on the activity of the compounds. It seemed that the addition of more sugar units inversely affected the antioxidant activity of the cyanidin [322]. Similar conclusions were drawn for the glycosylation effects on the antioxidant potentials of the cyanidin-based structures as observed on the RBCs in presence of AAPH-induced oxidative stress conditions [323]. The level of ROS was significantly decreased as observed for the aglycone, cyanidin, as well as the glycosides, cyanidin-3-O–arabinoside, cyanidin-3-O-galactoside, and cyanidin-3-5-O-di-glucoside. However, the cyanidin-3-O-rutinoside and cyanidin-3-O-β-glucoside have showed no significant effects [323]. The importance of 3-OH group of the anthocyanidins, and corresponding anthocyanins for the antioxidant activity of these compounds has been demonstrated by Ali et al., using eight different anthocyanidins, and seven anthocyanins, as well as two synthetic products based on 4′-hydroxy flavylium compounds, which were examined for their scavenging activity by the DPPH and ABTS (2,2′-azino-bis(3-ethylbenzothiazoline-6-sulphonic acid)) radicals, as well as their reducing power for the ferric ions using FRAP assay [153]. It was found out that the presence of 3-OH group in anthocyanidins, and anthocyanins, significantly enhanced the hydrogen atom release, and scavenged the DPPH and ABTS radicals, which was attributed to the stabilization by the anthocyanidin’s semi-quinone-like resonance. The reduction activity of the anthocyanidins was also improved by the presence of 3-OH group. However, both the anthocyanidins, and their respective anthocyanins, exhibited similar trends, and close scavenging and reducing activities [153]. 

As mentioned before, some anthocyanins metabolites are highly active as an antioxidant agent and mostly play major role in the known activity of anthocyanins. The major anthocyanins metabolic product, protocatechuic acid, has been reported for its powerful antioxidant and anti-fibrotic activities associated with its reducing capacity to the liver fibrosis pathological factors, e.g., transforming growth factor-β1 (TGF-β1), and connective transforming growth factor (CTGF) [324]. Other major anthocyanins metabolites, such as, gallic aid, syringic acid, and 3-O-methylgallic acid are also part of the anthocyanins bioactivity, and have been reported for their potential antioxidant, hepatoprotection, as well as anti-hepatocellular carcinoma [325,326,327].

## 17. Commercialization and Anthocyanins-Based Products in the Market

Anthocyanins have multiple health benefits, and are found in a wide variety of plants parts, i.e., fruits, seeds, leaves, and roots. The anthocyanins have been reported for their strong anti-oxidant, anti-inflammatory, and other bioactivities importance from the health maintenance perspective. They are also reported for their hepatoprotective, chemo-protective, and cardio-protective effects [272]. The anthocyanins are also safe, produce no toxicity, and provide major health benefits [30]. According to the Market Data Forecast webpage, the market size of anthocyanins products are growing at an annual rate of 4.6%, and the total market size of the anthocyanins-based products is expected to reach 388 million USD by 2026 [328]. In addition, the anthocyanins-based applications are also extended to the beverages, food supplements, and personal care based products, and their industries. Therefore, the anthocyanins-rich plants have been of much interest for the food, immunity booster, and medicinal use-based healthcare markets, and have been promoted as food and drug additives with several other drugs, herbal extract, and the anthocyanins itself in certain dosage forms, i.e., liquids, tablets, and capsules [272,329]. Hence, the herbal pharmaceutical preparations containing anthocyanins are socially accepted and reputed world-wide, is popular with masses, especially through Internet commercial websites, and also as part of over the counter (OTC) drugs in community pharmacies. The cranberry, bilberry, black berry, blood orange, raspberry, chokeberry, elderberry, mulberry, and blueberry-based products as extracts, powder, tablets, caplets, capsules are available in the market (Table 4), and are sold for the purpose of weight loss, as antioxidant, immunostimulant, general health well-being, and for urinary tract health. A number of criteria, including standard brands and their value, usefulness, certification, quantitative specifications, customer ratings, seller ranks, customer reviews, durability, and least negative ratings have been considered in listing the commercial products. A total of 57634 product reviews were recently (2/2022) analyzed by Clark [330] and forms part of the current listing. 

## 18. Conclusions and Prospects

Anthocyanins, the colored pigments of the plant kingdom, distributed in all parts of the plants, and especially appealing, and indicating the fruit ripening, have been in use as traditional medicament since ages for various ailments including liver disorders. The structural variations of the anthocyanins class are tremendous with over 700 products categorized based on their 27 major structural types which have been sub-divided into sections with the positional substitutions, substitution types (hydroxy, methoxy, and acetoxy), sugar variations, as well as into acetylated and non-acetylated anthocyanin products. The products have been analyzed through the conventional as well as state-of-the-art spectro-analytical and chromatographic techniques for their extractions, isolation, purification, and identification. Several health benefits and medicinal applications of the anthocyanins and anthocyanidins based plants extracts have been reported, probably owing to their potential antioxidant roles in the ingested food matrix. The anthocyanins have been used as protective agents against heart, liver, and brain disorders, as well as consumed for general health-well-being, obesity, stress, anti-inflammation, which is also attributed to their strong antioxidant properties. The strong anti-oxidant potential of the anthocyanins has been considered to be the reason for their strong biological activity of different pharmacological classes. The health benefits have led the nutritional bodies recommending the daily intake of the anthocyanins as a mixture of products, wherein single pure anthocyanin product has not been observed to be of similar potency and advantage as the mixture. There have been no toxicity reported for anthocyanins based on the daily intake in humans. The products stability, pH-based structural alterations, and accumulation in plants have made these pigments of immense commercial value. The bioactivity against liver disorders, prophylactic hepatoprotection, activity against liver carcinoma, and hepatobiliary cancers, as well as prolonging the life of the diseased liver have been attributed to the anthocyanins mixture’s action on the liver. Multiple biomechanistics routes have also been discovered behind the bioactions of the anthocyanins, including for the liver disorders. Free-radical scavenging, anti-inflammatory properties, anti-lipid peroxidation capability, changes in liver biomarkers, up and down-regulation of the genes and their factors, approach and increase in signal transduction, inflammatory cytokines, signaling involvements, and activation of the physiological pathways have been suggested to be participating. New nano-formulations, synthetic products, and bioavailability enhancements strategies developments have led to wide-spread commercialization of the anthocyanins products which has been adopted to their source-plants in industrial agriculture, and crop and its quality management of the anthocyanin products as marketed commodity for the use by common man, and to elderly for age-related diseases, and for the adults and children as food supplements. Prospects exists in synthetic analogs development, formulations, and yields enhancements from the source plants. Developments in extraction methodology, quantification and quality control of the products, yields enhancements through biotechnical advances, and structure–activity relationships of the anthocyanin products, diseases and the receptor entanglements for better drug leads are also desirable. The efforts to meet the future market demands through newer strategies in production, storage, and stability of the anthocyanin products are inevitable.

## Figures and Tables

**Table 1:** Plants and their parts used in treatment of various diseases and the identified anthocyanin contents.

Plant’s Name	Folklore Medicinal Uses, Other than Liver Disorders	Plant Parts Used	Major Identified Anthocyanins	Refer
*Hibiscus sabdariffa*	Hypertension, pyrexia	Calyx, Epicalyx	Cyanidin-3-O-β-glucoside, and delphinidin-3-glucoside	[175,176]
*Cichorium intybus*	Inflammation	Leaves	Cyanidin-3-*O*-(6″-malonyl-β-glucopyranoside)	[177]
*Garcinia indica*	Male digestion, flatulence, and constipation.	Fruits	Cyanidin-3-O-β-glucoside, and cyanidin-3-O-sambubioside	[178]
*Raphanus sativus*		Roots	Pelargonidin derivatives	[179]
*Morus* alba (Mulberry), & other species	Cardiovascular diseases, nephritis, thirsty, constipation	Fruits	Cyanidin-3-O-rutinoside, cyanidin-3-O-glucoside	[180,181]
*Cornus mas* (cornelian cherry)	Diabetes, diarrhea, fevers, rheumatic complains, skin diseases and urinary tract infections	Fruits	Cyanidin-3-O-galactoside, pelargonidin-3-O-galactoside, delphinidin-3-O-galactoside, cyanidin-3-O-rutinoside, pelargonidin-3-O-glucoside, pelargonidin-3-O-rutinoside, pegonidin-3-O-glucoside	[182]
*Lannea microcarpa*	Scurvy, rickets and cough.	Fruits	Cyanidin-3-O-(2-O-β-D-xylopyranosyl)-β-D-galactopyranoside, and cyanidin-3-O-β-D-galactopyranoside.	[183]

**Table 2:** Anthocyanins modes of action as liver-protecting agents against induced liver injuries.

Anthocyanins	Experimental Protocol	Mode of Action	Refer
Cyanidin-3-O-β-glucoside	In vivo CCl_4_-induced liver damage in mice and in vitro H_2_O_2_-induced oxidative stress in HepG2 cells apoptosis	Enhance the antioxidant enzymes activities and upregulating Nrf2-antioxidant pathway.	[156]
Delphinidin	In vitro H_2_O_2_-induced oxidative stress in HepG2 Cells	Enhance the expression of Nrf2 and promoted Nrf2 nuclear translocation. Increase expression of antioxidant protein HO-1 (Nrf2-related phase II enzyme heme oxygenase-1). Alleviate the reduction of Nrf2 protein levels and the accumulation of intracellular ROS levels in Nrf2 knockdown HepG2 cells.	[288]
Mixture of cyanidin-3-O-β-glucoside, delphinidin-3-O-rutinoside, and malvidin-3-O-galactoside	In vivo CCl_4_-induced human embryonic-liver (L-02) cells toxicity	Reduce the percentage of hypo-diploid cells and decrease in caspase-3 protein expression	[289]
Cyanidin-3-O-β-glucoside, and peonidin-3-O-glucoside	In vitro human embryo non-malignant liver tissue cell line (L-02). Hepatoprotection	Exhibited higher cell viability, decreased aminotransferase activity and enhanced cellular antioxidant status. Furthermore, Cy-3-G showed much stronger hepatoprotective activity than Pn-3-G at the same concentration.	[186]

**Table 4:** Major suppliers of anthocyanins-based nutraceuticals, and food-supplements.

No.	Supplier/Company	Product’s Name	Plants/Declared Anthocyanins	Company’s Address
1.	Solgar	Bilberry Berry Extract	*Vaccinium myrtillus*	https://www.solgar.com/
2.	Sports Research	Blueberry Concentrate	*Vaccinium cyanococcus*	https://sportsresearch.com/
3.	Life Extension	Tart Cherry Extract	*Prunus cerasus*	https://www.lifeextension.com/
4.	Navitas Organics	Organic-Maqui Powder, Tart Berry	Anthocyanins contents	https://navitasorganics.com/
5.	Nature’s Plus	Black Cherry	0.8 % Anthocyanins	https://naturesplus.com/
6.	NOW Foods	Super Antioxidants	Bilberry Extract	https://www.nowfoods.com/
7.	Natural Factors	BlueRich Super Strength	Blueberry concentrate	https://naturalfactors.com/en-ca/
8.	Nature’s Truth	Bilberry	Bilberry concentrate	https://naturestruth.com/
9.	Vision Alive		Bilberries, blueberries, from black currant, Maqui berry, saffron, and astaxanthin	https://visionalive.net/products.html
10.	New Nordic	Blueberry Eyebright	Conc. blue berry extract	https://newnordic.com/
11.	Vitabiotics	Bilberry, Lutein	500 mg Bilberry	http://www.vitabiotics.com/
12.	Sambucol	Syrup Black-Elderberry	Elderberry extract 1.9/10 mL	https://sambucol.co.uk/
13.	Fairvital	Aronia Capsules	20% anthocyanins	https://www.fairvital.com/en/index
14.	TruNature	Blueberry	Blueberry extract 1000 mg	http://trunature.com/
15.	TruNature	One per day Cranberry	Cranberry 650 mg	http://trunature.com/
16.	Life Extension	Optimized Cran-Max	Cranberry whole fruit conc.	https://www.lifeextension.com/
17.	Life Extension	Garcinia HCA	*G. cambogia* fruits extract	https://www.lifeextension.com/
18.	Georgia’s Natural	Organic Carrot Juice	Purple carrot juice 99%	https://georgiasnatural.com/ge/intro
19.	Santarome	Bio Black Radish	Black radish juice	https://en.santarome.fr/
20.	PipingRock	Hibiscus Flower	*H sabdariffa* 11 mg in extract	https://sa.pipingrock.com/brand/piping-rock
21.	Swanson	Hibiscus Flower	*Hibiscus sabdariffa*	https://swansoneurope.com/
22.	Bluebonnet	Skinny Garcinia	*Garcinia* fruit rind extract	https://bluebonnetnutrition.com/
23.	Ceregumil	Vid-Master Plant	*Vaccinium corymbosum*	https://sa.carethy.net/ceregumil
24.	Source Naturals	Pomegranate Extract	*Punica granatum* extract	https://www.sourcenaturals.com/
25.	AllMax	Nutrition Liver D-Tox	Artichoke extract	https://allmaxnutrition.com/
26.	PureClinica	Anti-oxidant Bilberry	Bilberry extract	https://pureclinica.com/
27.	SYM Nutrition	Bilberry	25% Anthocyanins	https://symnutrition.com/
28.	Vision Smart Center	Blackcurrant Anthocyanins	210 mg Anthocyanins, cyanidin, and delphinidin based	https://visionsmartcenter.com//
29.	Designs for health	Grape Seeds and Grape Skin	Anthocyanins and grapes’ polyphenol anti-oxidant blend	https://designsforhealth.com/
30.	Berry Supplements	Anti-oxidant Nutritional Supplement	Aronia berry powder	https://bulksupplements.com/
